# Albumin Nanoparticles Improve Colistin Performance Against Hetero- and Full-Resistant Clinical *A. baumannii*: A Mechanistic Study

**DOI:** 10.3390/antibiotics15040410

**Published:** 2026-04-17

**Authors:** Sara Scutera, Viviana Cafiso, Giulia Vigna, Monica Argenziano, Eleonora Chines, Antonio Curtoni, Matteo Florio Furno, Giovanna Cristina Varese, Chiara Scarpa, Ilario Ferrocino, Stefania Raimondo, Gabriele Bianco, Roberta Cavalli, Tiziana Musso

**Affiliations:** 1 Department of Public Health and Pediatrics, University of Turin, 10126 Torino, Italy; giulia.vigna@unito.it (G.V.); antonio.curtoni@unito.it (A.C.); tiziana.musso@unito.it (T.M.); 2 Department of Biomedical and Biotechnological Sciences, University of Catania, 95123 Catania, Italy; v.cafiso@unict.it (V.C.); eleonora.chines01@universitadipavia.it (E.C.); 3 Department of Drug Science and Technology, University of Turin, 10125 Torino, Italy; monica.argenziano@unito.it (M.A.); chiara.scarpa@unito.it (C.S.); roberta.cavalli@unito.it (R.C.); 4 Department of Public Health, Experimental, and Forensic Medicine, University of Pavia, 27100 Pavia, Italy; 5 Department of Life Sciences and Systems Biology, University of Turin, 10123 Torino, Italy; matteo.floriofurno@unito.it (M.F.F.); cristina.varese@unito.it (G.C.V.); 6 Department of Agricultural, Forest and Food Science, University of Turin, 10095 Grugliasco, Italy; ilario.ferrocino@unito.it; 7 Department of Clinical and Biological Sciences and Neuroscience Institute Cavalieri Ottolenghi (NICO), University of Turin, 10043 Orbassano, Italy; stefania.raimondo@unito.it; 8 Department of Experimental Medicine, University of Salento, 73100 Lecce, Italy; gabriele.bianco@unisalento.it

**Keywords:** *Acinetobacter baumannii*, colistin, albumin nanoparticles, heteroresistant isolates

## Abstract

**Background:** Colistin (Col) resistance and heteroresistance in extensively drug-resistant (XDR) *Acinetobacter baumannii* severely limit therapeutic options. We investigated the activity and mechanism of human albumin nanoparticles (haNPs) as colistin potentiators against genetically characterized clinical isolates. **Methods:** Sixteen clinical isolates were analyzed. Col MICs were determined by broth microdilution, and heteroresistance by population analysis profiling. Potentiation of Col activity was assessed using both Col-loaded haNPs (Col/haNPs) and free Col co-administered with empty haNPs, alongside the proton motive force (PMF) uncoupler carbonyl cyanide 3-chlorophenylhydrazone (CCCP). Assays included checkerboard synergy (FICI), membrane potential analysis (DiOC_2_(3)), intracellular Col quantification (UPLC–MS/MS), zeta potential measurements, transmission electron microscopy (TEM), protein leakage, and ROS detection. **Results:** Heteroresistance was detected in 9/16 isolates. Col/haNPs reduced Col MICs by 4–64-fold in resistant strains and shifted MICs to ≤2 mg/L in most heteroresistant isolates. Empty haNPs displayed no intrinsic antibacterial activity yet selectively potentiated Col, with strong synergy (FICI down to 0.035). Membrane depolarization and increased intracellular Col accumulation under haNP-treated conditions paralleled the effects of CCCP, indicating that haNPs elicit a CCCP-like functional response. These findings are compatible with perturbation of membrane energetics and possible downstream effects on PMF-dependent transport processes. TEM and surface charge analyses supported direct nanoparticle–envelope interaction and progressive membrane disruption. **Conclusions:** haNPs enhance Col activity across genetically diverse *A. baumannii* isolates, with particularly strong effects in heteroresistant strains. The combined effects of PMF modulation, increased intracellular drug availability, and envelope interaction provide a mechanistic rationale for the use of albumin-based nanoparticles, either as Col carriers or in combination with free drug, to overcome Col resistance and heteroresistance.

## 1. Introduction

*Acinetobacter baumannii* is a Gram-negative opportunistic pathogen and a major cause of hospital-acquired infections such as bacteremia, pneumonia, urinary tract infections, and wound infections. These infections predominantly affect critically ill patients in intensive care units (ICUs) and are associated with high mortality. Treatment is increasingly challenging due to the rapid emergence of strains resistant to carbapenems and multiple antimicrobial classes [1]. Consequently, therapeutic options for multidrug-resistant (MDR) and extensively drug-resistant (XDR) *A. baumannii* are extremely limited, with colistin (Col) often regarded as a last-resort therapy. However, the renewed reliance on Col has led to the worldwide emergence of Col-resistant strains, further narrowing treatment choices.

Col exerts its bactericidal activity primarily by binding to lipopolysaccharide (LPS), disrupting the outer membrane, and increasing permeability. Additional mechanisms, such as inhibition of key respiratory enzymes and induction of hydroxyl radical production, have also been proposed. Resistance to Col commonly arises through modifications of lipid A, including the addition of phosphoethanolamine (PEtN) mediated by mutations in the *pmrA*/*pmrB* genes (the *pmrCAB* operon), *eptA*, or plasmid-encoded *mcr* genes. Other mechanisms involve complete or partial loss of LPS due to mutations in the *lpxACD* operon. Moreover, efflux pumps are increasingly recognized as contributors to Col resistance. These systems, including the resistance-nodulation-division (RND), major facilitator superfamily (MFS), and multidrug and toxic compound extrusion (MATE) families, actively expel antibiotics and reduce intracellular drug accumulation [2,3,4]. Overexpression of efflux components and their regulators has been linked to both intrinsic and acquired resistance, and inhibition of efflux with compounds such as carbonyl cyanide m-chlorophenyl hydrazone (CCCP) has been shown to restore Col susceptibility [5].

Various antimicrobial nanoparticles and nanosized carriers have shown effectiveness in overcoming MDR by improving drug stability, bioavailability, and antibacterial potency [6].

Among organic biomaterials, albumin has emerged as one of the most versatile carrier proteins owing to its biocompatibility, non-immunogenicity, non-toxicity, low cost, and high drug-loading capacity. Albumin nanoparticles can also be easily functionalized through their charged surface groups, making them attractive candidates for drug delivery. Albumin-based nanoparticles have been widely explored in anticancer therapy [7,8]. More recently, their potential in antimicrobial applications has also attracted growing interest [9].

In our previous work [10], we developed Col-loaded human albumin nanoparticles (Col/haNPs), which have been shown to enhance antimicrobial and antibiofilm effects against resistant Gram-negative bacteria, suggesting that this formulation can lower the effective dose of Col required for bacterial inhibition, potentially reducing toxicity while improving therapeutic efficacy. Despite these promising findings, the precise mechanisms by which Col/haNPs counteract resistance in *A. baumannii* remain poorly understood.

Building on these findings, we investigated the activity of Col/haNPs against clinical isolates of *A. baumannii* with a characterized resistance profile. By combining microbiological assays, efflux activity evaluation, and nanoparticle–bacteria interaction, we aimed to clarify how haNPs influence bacterial physiology and restore Col susceptibility. Specifically, we compared encapsulated versus co-administered Col to define the carrier-specific role, providing a rational basis for nanoparticle-based strategies against MDR infections.

## 2. Results

### 2.1. Properties of Col-Loaded Chitosan-Coated Albumin Nanoparticles (haNPs)

Stable haNPs with average diameter of about 180 nm, low polydispersity index and positive surface charge (15 mV) due to the chitosan coating, were obtained. Col was efficiently loaded within the albumin matrix of haNPs, showing an encapsulation efficiency of about 98%.

### 2.2. Genotypically Characterized Col-Resistant Acinetobacter baumannii Strains

A total of 16 XDR *A. baumannii* clinical isolates were included in this study (Table 1).

Multi-locus sequence typing (MLST) was performed according to the Pasteur scheme for *A. baumannii*. Genomic phylogeny (gPhyl) identified four distinct lineages, as reported in Table 1 and illustrated by the whole-genome single nucleotide polymorphism (SNP)-based phylogenetic tree (Figure S1).

*A. baumannii* 5R, 6R, 7R, 8R, and 9R were grouped in gPhyl lineage-I; 3R, 4R, and the C, F, and G strains in gPhyl lineage-II; and 1R, 2R, B, D, and E strains in gPhyl lineage-III [11]. Only isolate A belonged to international clone IV. According to the Pasteur Institute MLST database, all *A. baumannii* strains were assigned to sequence type ST2, except for strain A (ST636) and strain 1R (ST187).

K and OC loci, respectively encoding capsular and outer core polysaccharide biosynthesis, were typed among the 16 isolates sequenced (Table 1). Seven different KLs were found, namely KL9 (from strains 5R to 9R), KL 22 (strains B, D, E, 1R, and 2R), KL2 (strains F and G), KL3 (strain 3R), KL7 (strain C), KL28 (strain 4R), and KL40 (strain A). The sequences were also examined for the presence of the reported forms of the OC locus [12]. OCL1 is the proposed ancestral type, and it was found in 10 genomes, three of which carry the two different variants OCL1c (strains F and G) and OCL1d (strain C), previously described. OCL3 was found in five strains (B, D, E, 1R, and 2R), while OCL2 was found only in one strain (A) [13,14,15].

Resistome analysis revealed that isolates B, D, and E contained an identical set of genes responsible for the resistance to β-lactams and aminoglycosides (*blaADC-25*, *blaOXA-23*, *blaOXA-82*, *aadA2*, *ant(2″)-Ia*, *aph(3′)*-*VIa*), also found in strains 1R and 2R. Isolates A and 5R to 9R share *blaADC-25*, *blaOXA-66*, and *blaOXA-72* but carry different aminoglycoside determinants. Almost the same resistome was observed in isolates C, F, G, 3R, and 4R (*blaOXA-23*, *blaOXA-66*, *blaADC-25*, *aph(6)-Id*, *aph(3″)-Ib*), except for the presence of specific genes in some strains: 3R presented *blaTEM-1D* and *armA* alongside *aph(3′)-Ia*; strain C *ant(3″)-Ia* and *aph(3′)-Ia*; strains F and G presented the *armA* gene. All resistome analysis is presented in Table S1 and [11].

### 2.3. Col Resistance Determination and Efficacy of Col-Loaded Albumin Nanoparticles

Col susceptibility was assessed using the broth microdilution method, and the minimum inhibitory concentration (MIC) values are presented in Table 2. In 10 independent MIC determinations, seven strains consistently exhibited high and stable Col MICs, ranging from 40 to >160 μg/mL, indicating a fully resistant phenotype.

**Figure 1:**
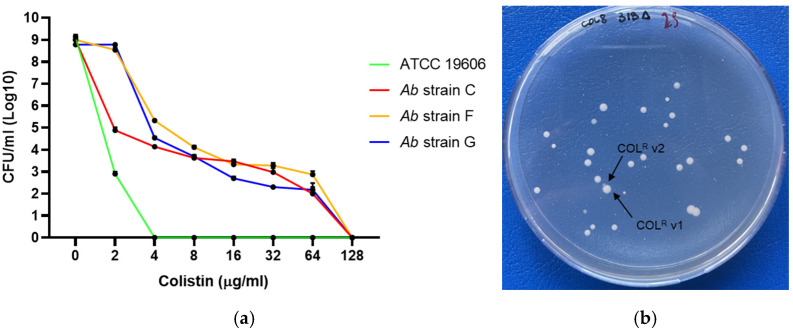
(**a**) PAP assay for the C, F, and G *A. baumannii* strains. (**b**) Representative morphologies of the COL-R *A. baumannii* variants detected in strain G on 8 μg/mL Col-agar plates.

Nine clinical isolates were characterized as Col-heteroresistant, including six control strains identified in previous studies [11] and three newly isolated strains characterized in the present work. Heteroresistance for the strains C, F, and G was confirmed by PAP, which revealed the presence of resistant subpopulations capable of growing in the presence of up to 64 μg/mL Col sulphate (Figure 1). On Col-supplemented agar plates (8 or 16 μg/mL), two distinct resistant subpopulations, referred to as COL-R variant 1 and variant 2, were identified in strains C, F, and G, as previously described for strains 4R, 5R, 7R, and 9R [11]. COL-R variant 2 exhibited higher Col MICs than variant 1 in all three strains. Specifically, 16-fold, 8-fold, and 2-fold increases were observed in strains G (256 vs. 16 μg/mL), C (128 vs. 16 μg/mL), and F (64 vs. 32 μg/mL), respectively.

The antimicrobial activity of Col/haNPs was evaluated against all Col-resistance (COL-R) strains. Col/haNPs significantly reduced the MIC of Col by 4- to 64-fold in all Col-resistant *A. baumannii* isolates. Notably, in all heteroresistant strains, except strain G, Col/haNPs reduced MICs to below 2 mg/L, the European Committee on Antimicrobial Susceptibility Testing (EUCAST) breakpoint for free Col (Table 2). Col/haNPs also significantly lowered MIC values in Col-S strains. A comparison between the concentrations of Col encapsulated in haNPs (based on the MIC values of the nanoparticle formulation) and those of free Col revealed that substantially lower amounts of the antibiotic were required to inhibit bacterial growth. Specifically, MIC values were reduced 4- to 8-fold in *A. baumannii* ATCC19606, ATCC17978, ACICU, and in three susceptible clinical strains (*Ab* 1S, *Ab* 2S, and *Ab* 3S), as shown in Table 2. As expected, haNPs alone did not exhibit antibacterial activity against all these strains, as previously reported [10].

### 2.4. Genotypic and Phenotypic Characterization of Col Resistance in Clinical A. baumannii Isolates

To explore the relationship between resistance mechanisms and NP antimicrobial activity, we characterized the genomic Col resistance profile of bacterial strains A–G. In *A. baumannii*, Col resistance is primarily mediated by two LPS-related mechanisms: complete LPS loss, due to substitutions, deletions, or ISAba1 insertions in *lpxA*, *lpxC*, or *lpxD*, and lipid A modification via phosphoethanolamine addition, driven by either the PmrAB system or the MCR-1 enzyme.

**Figure 2:**
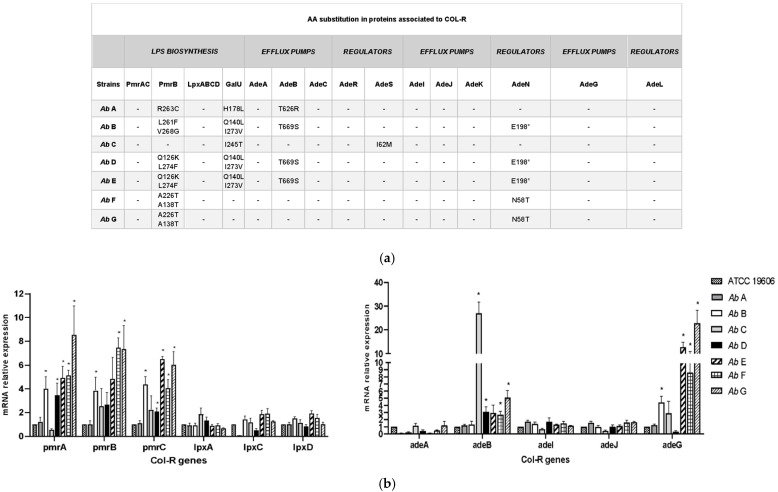
(**a**) AA changes in COL-R strains relative to Col resistance and determined by a moderate impact effect (MI) on non-synonymous single nucleotide polymorphisms (nsSNPs). * STOP CODON: c. 592G>T|p.Glu198* protein AdeN of 198AA instead of 217AA. (**b**) Relative expression of COL-R-associated genes in *A. baumannii* COL-R strains compared to the reference strain ATCC19606. *rpoB* was used to normalize expression levels. Data were calculated using the ΔCT method and expressed as the mean ± SEM of three independent experiments. * <0.05 versus ATCC19606 by Student’s *t*-test.

Whole-genome sequencing (WGS) analysis of the isolates revealed the absence of plasmid-mediated Col resistance genes (*mcr-1* to *mcr-10*). Amino acid substitutions in the PmrB protein were identified in all strains, with the exception of strain C. Specifically, non-synonymous single nucleotide polymorphisms (nsSNPs) with a predicted moderate impact (MI), resulting in different amino acid changes, were detected (Figure 2a). Strains F and G shared the same substitutions (A138T and A226T), whereas strains D and E harbored the Q126K and L274F substitutions. Strain B displayed the L261F and V268G substitutions, while strain A carried the R263C substitution in PmrB.

No mutations were found in PmrAC and Lpx ABCD among the seven isolates. Diverse MI nsSNPs in GalU, a key enzyme involved in the synthesis of LPS and capsular structures [16], were found in all the strains except F and G (Figure 2a).

Analysis of *pmrABC* expression revealed that *pmrA*, *pmrB*, and *pmrC* levels were statistically higher in Col-R isolates B, F, and G vs. strain ATCC 19606, while strain A showed expression levels comparable to those of the reference. In contrast, no significant differences were observed in the expression of *lpxA*, *lpxC*, or *lpxD* between the tested isolates and the reference strain (Figure 2b, left panel).

In addition to previously described mechanisms, the active efflux of Col through membrane transport systems represents another important contributor to resistance in *A. baumannii* [4,17]. The protein sequences of the efflux pumps genes *adeA*, *adeB*, *adeC*, *adeI*, *adeJ*, *adeK*, and *adeG* from the RND family and their respective pump regulators AdeRS, AdeN, and AdeL were studied. In AdeB, two different amino acid substitutions, T626R and T669S, were found. T669S was harbored by strains B, D, and E, and T626R was detected only in strain A. The substitution I62M in the regulator AdeS, previously detected in one tigecycline-resistant isolate [18], was present in strain C. No substitutions were found in AdeIJK, AdeG, and its regulator AdeL. In AdeN, the N58T mutation, previously linked to multidrug resistance [19], was detected in strains F and G, while the E198* nonsense mutation, resulting in a premature stop codon and previously associated with tigecycline resistance [20], was found in strains B, D, and E (Figure 2a).

We next evaluated the expression of efflux pump genes *adeA*, *adeB*, *adeG*, *adeI*, and *adeJ*, from the RND family, in Col-R strains, comparing them with the reference strain ATCC 19606.

No major differences were observed in the expression of *adeA*, *adeI*, and *adeJ* among the tested strains. In contrast, *adeB* expression was significantly elevated in strains C, D, F, and G compared to the reference strain ATCC 19606, with the highest levels detected in strain C. A higher expression for *adeB* was also observed in strain E. *AdeG* expression was significantly upregulated in isolates B, E, F, and G, whereas strains A and D showed levels comparable to or lower than those of the reference (Figure 2b, right panel).

**Figure 3:**
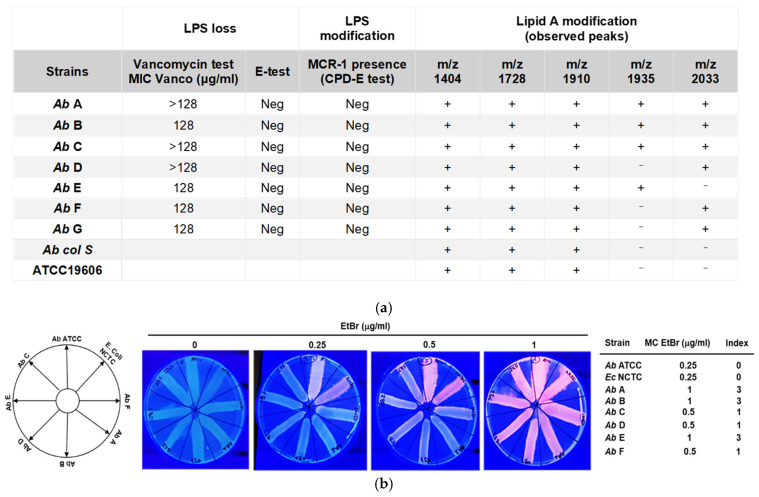
(**a**) LPS loss in *A. baumannii* COL-R strains was evaluated by sensitivity to vancomycin (increased in the absence of LPS) and growth pattern in the presence/absence of Col by E-test. MCR1-mediated resistance was identified using the CPD-E test, in which MCR1-expressing strains show an increased diffusion halo in the presence of EDTA. Lipid A structural analysis of *A. baumannii* isolates compared to an *A. baumannii* COL-S strain and ATCC19606 was performed using the MALDIXin test. (**b**) An EtBr-agar cartwheel assay was performed for the *A. baumannii* COL-R clinical strains, *E. coli* NCTC, and the reference strain *A. baumannii* ATCC19606, as comparative control. Cartwheel pattern for the swabbing of the bacterial strains tested, and the relationship between MCEtBr and efflux activity is shown. Fluorescence was observed at 1 μg/mL of EtBr for all the strains, while no fluorescence was present at 0.25 μg/mL for all clinical strains.

LPS loss was phenotypically assessed by evaluating vancomycin susceptibility using MIC testing, as this glycopeptide cannot cross an intact LPS-containing outer membrane [21,22]. All seven *A. baumannii* strains tested were resistant to vancomycin (MIC ≥ 128 µg/mL), indicating that none exhibited an LPS-deficient phenotype. An E-test assay with Col was performed on the same isolates to assess the presence of LPS-deficient strains. Such strains typically display a Col-dependent phenotype in zones where Col is present at concentrations below the MIC of the organism [23]. None of the tested isolates exhibited this characteristic phenotype. In addition, a phenotypic screening for plasmid-mediated Col resistance was carried out using the CPD-E test. No significant MIC reduction was observed in the presence of EDTA in any of the *A. baumannii* strains, suggesting the absence of MCR-1-associated resistance [24] (Figure 3a). 

Modifications of lipid A, frequently involving the addition of (pEtN) and/or galactosamine (GalN), have been associated with Col resistance in *A. baumannii* [25]. To investigate this mechanism, the lipid A profiles of COL-R isolates (n = 7) were analyzed by MALDI-TOF MS and compared with those of *A. baumannii* ATCC 19606 and one COL-S clinical strain. The lipid A spectrum of *A. baumannii* ATCC 19606 and all clinical strains showed three major peaks corresponding to bis-phosphoryl hepta-acylated lipid A (*m*/*z* 1910), bis-phosphoryl hexa-acylated lipid A (*m*/*z* 1728), and bis-phosphoryl tetra-acylated lipid A (*m*/*z* 1404). In contrast, COL-R strains displayed two additional peaks, absent in the COL-S spectra, at *m*/*z* 1935, corresponding to the addition of pEtN to the phosphate group at position 4′ of native LPS, and at *m*/*z* 2032, corresponding to the addition of pEtN to the phosphate group at position 1 of native lipid A (Figure 3a and Figure S2) [26].

To qualitatively assess the involvement of efflux pumps, the cartwheel test was performed using different concentrations of ethidium bromide (EtBr), ranging from 0.125 to 2 µg/mL. After incubation, bacterial masses exhibiting different levels of fluorescence were observed, reflecting their varying ability to extrude EtBr. Figure 3b shows the method and the results obtained with EtBr concentrations ranging from 0.25 µg/mL (minimum concentration of EtBr, MCEtBr), which produced fluorescence in the reference strain ATCC19606 and in *E. coli* NCTC13846 MCR-1 positive, to 1 µg/mL, at which fluorescence was detected in all tested strains. The efflux capacity of the XDR clinical strains was ranked relative to the reference strain by calculating the efflux activity index, as described in the Section 4. The calculated index enabled a relative comparison of efflux activity among the XDR strains, consistent with what is expected for characterized XDR clinical isolates. Overall, EtBr efflux activity was detectable in all tested species, although at different levels.

### 2.5. CCCP-Comparable Potentiation of Colistin by haNPs

The effect of efflux pump inhibitors (EPIs), such as oxidative phosphorylation uncoupler CCCP, on reversing Col resistance in *Acinetobacter* and other Gram-negative pathogens (*Klebsiella*, *Pseudomonas*) highlights the role of efflux pumps in Col resistance mechanisms [5,27,28,29]. We therefore explored the effect of CCCP on the Col MIC of our strains. All tested isolates showed a >4-fold reduction in Col MIC in the presence of CCCP, with the largest decrease (>1026-fold) observed in strains B, D, and E (Table 3). All isolates grew in the presence of CCCP at the tested concentration (10 mg/L in DMSO) when administered without Col, confirming that CCCP alone did not exert antibacterial activity at this dose, consistent with prior observations [5]. Because nanoparticles have also been proposed to act as EPIs [30], we investigated whether empty haNPs could reduce Col MIC. In strains A, B, C, D, F, and G, haNPs (8–16 µM as albumin concentration) reduced Col MIC by ≥16-fold, with strains F and G showing reductions exceeding 512-fold. Strain E exhibited a 4-fold decrease, and the Col-susceptible reference strain ATCC 19606 showed a 16-fold decrease. Moreover, we found that the addition of haNPs as well as CCCP did not significantly alter the MIC (>4-fold reduction) of the tested strains to other antibiotics such as chloramphenicol and ciprofloxacin (Table S2). The reduction in Col MIC by haNPs is consistent with a CCCP-comparable potentiating effect on Col susceptibility.

### 2.6. Evaluation of the Combined haNPs and Antibiotic Treatments

To determine whether there is direct interaction between haNPs and Col, the surface charge of haNPs was investigated both before and after incubation with Col by DLS analysis. A decrease of about 30% in the haNPs’ zeta potential (15 mV and 9.5 mV for haNPs and haNPs + Col, respectively) was observed after incubation with Col. This decrease can be ascribed to electrostatic interactions between haNPs and Col, resulting in protein conformational changes and Col adsorption on haNPs [31,32].

The molecular interactions between haNPs and Col were studied using Fourier-transform infrared spectroscopy (FTIR) (Figure S3). The Col spectrum showed a broad band in the 2000–4000 cm^−1^ region related to hydrogen bonding and characteristic peaks at 1645 and 1538 cm^−1^ related to the Amide I and Amide II bands. The shift and change in peak intensity in the spectrum of haNPs + Col compared to Col supported the occurrence of molecular interactions between the drug and the haNPs. These interactions could be hydrogen bonding, van der Waals, and electrostatic interactions. Moreover, FTIR analysis of Col/haNPs previously confirmed the incorporation of the drug inside the albumin matrix, as the characteristic bands of Col were not detected.

**Figure 4:**
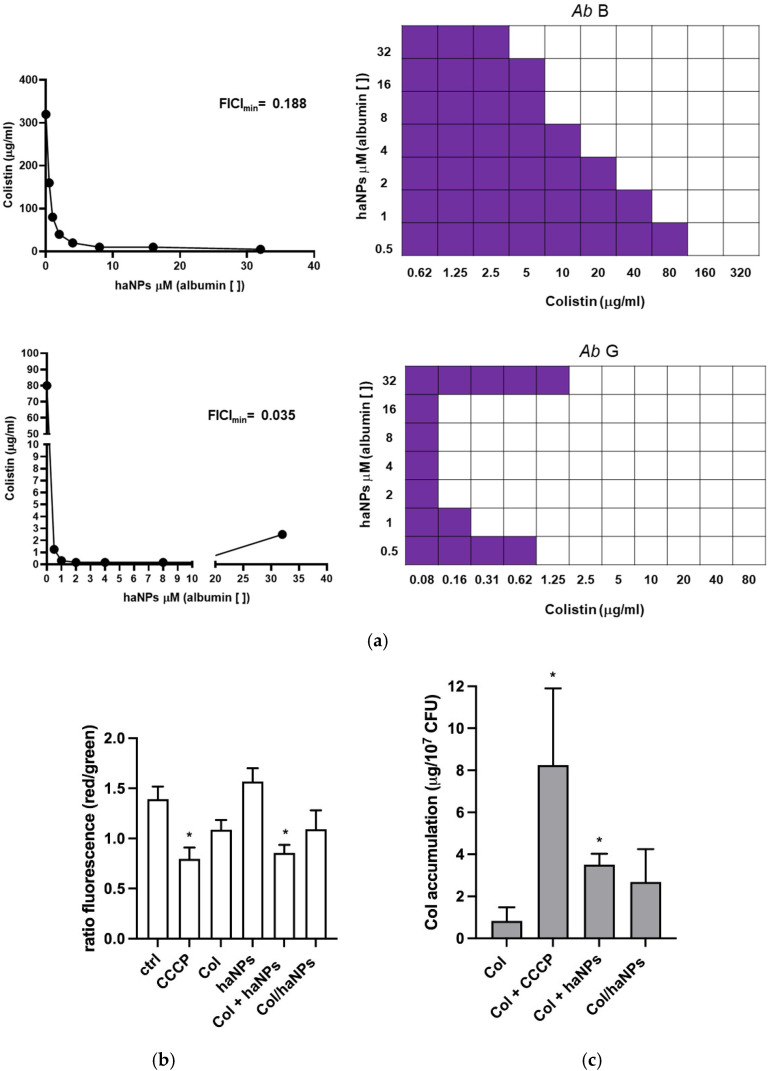
(**a**) Representative images of checkerboard assays and isobolograms for evaluation of synergism between Col and blank haNPs against *A. baumannii* clinical strains B and G. The purple wells indicate typical bacterial growth, while white wells indicate inhibited bacterial growth (right panels). The isobolograms (left panels) illustrated the results of the checkerboard assay and the FICI values, showing the synergistic curve. The x axis of the isobologram represents the dose of haNPs as albumin concentration (μM), and the y axis the dose of Col (μg/mL). (**b**) The effect of haNPs alone or in combination with Col and of Col/haNPs (1 × MIC) on membrane potential was evaluated by FACS analysis. CCCP (100 μg/mL) is used as a positive control. A significant disruption of bacterial membrane potential based on the reduction in the red/green fluorescence ratio in the DiOC_2_(3) -probed cells as mean fluorescence intensity (MFI). The bacterial membrane potential of the full-resistant strains A and E is shown as mean ± SEM of five independent experiments. * <0.05 versus untreated bacteria (ctrl) by Student’s *t*-test. (**c**) The intracellular accumulation of Col in bacteria treated with Col (1 × MIC), Col + haNPs (16 μM albumin concentration), Col + CCCP (10 μg/mL), Col/haNPs (1 × MIC) was evaluated using HPLC assay. Mean ± SEM of Col expressed as micrograms in 10^7^ bacteria in three independent experiments. * <0.05 versus free Col-treated bacteria by Student’s *t*-test.

In addition, the interaction between Col and haNPs was evaluated by checkerboard microtiter assay, with the FICI calculated using two strains (B and G), selected as representative examples of Col full- and heteroresistance (B and G, respectively) [33]. As expected, haNPs alone did not exhibit antimicrobial activity; however, even at low concentrations, combination with Col enhanced their antibacterial activity. For strain B at higher haNP concentrations (8–16 μM), the Col MIC was reduced to 10 μg/mL, corresponding to a 32-fold decrease (Figure 4a and Table S3), indicating a dose-dependent potentiation of Col by haNPs. Moreover, for strain G, the Col MIC was reduced 513-fold compared to the MIC of Col alone, using haNPs at concentrations ranging from 2 to 16 μM (Table S3). Isobologram analysis revealed strong synergism for strains B and G, with FICI values up to 0.188 and 0.035, respectively (Figure 4a). Compared with the MIC of Col/haNPs, a reduction from 2.5 μg/mL (Table 2) to 0.31 μg/mL (below EUCAST breakpoint), in the presence of 1 μM of haNPs (as albumin concentration), was observed for strain G, indicating a potential use of haNPs in association with free Col.

### 2.7. haNPs/Col Induces Changes in Bacterial Membrane Potential

Because efflux pump activity depends on the proton motive force, which is closely linked to membrane polarization, the effect of haNPs on the bacterial membrane was investigated. Flow cytometry analysis with DiOC_2_(3) was performed to evaluate the impact of Col, haNPs alone, and Col and haNPs combined or loaded (Col + haNPs and Col/haNPs) on bacterial membrane potential of two representative full-resistant strains (A and E). CCCP was used as a positive control, as it acts as a protonophore that dissipates the electrochemical gradient and causes rapid membrane depolarization. In this assay, DiOC_2_(3) emits red fluorescence in polarized cells and green fluorescence in depolarized cells, such that a reduction in the red/green fluorescence ratio reflects membrane depolarization [34]. As shown in Figure 4b, untreated control bacteria exhibited a high red/green ratio consistent with an intact membrane potential. In contrast, a marked decrease in this ratio was observed upon treatment with Col + haNPs, to an extent comparable to CCCP, while Col alone and Col/haNPs produced a more moderate depolarizing effect. These results show that the synergistic activity of Col and haNPs is associated with disruption of bacterial membrane potential, a phenomenon that could affect PMF-dependent transport processes, including efflux activity [35,36].

### 2.8. Enhanced Intracellular Accumulation of Col Induced by CCCP and haNPs

To further investigate the role of efflux pumps, we quantified intracellular Col accumulation using UPLC–MS/MS. Bacteria were exposed to Col alone and Col in the presence of CCCP or haNPs. The combination of Col with either CCCP or haNPs resulted in a significant increase in intracellular Col concentration compared to bacteria treated with free Col. When Col was delivered as Col/haNPs, the MIC was achieved at a lower nominal Col concentration, and intracellular Col showed only a modest, non-significant upward trend, an observation compatible with formulation-dependent kinetics (carrier-associated drug and slower release) and the limitation of a single-timepoint measurement for encapsulated formulations (Figure 4c).

### 2.9. Zeta Potential Measurements of A. baumannii upon Treatment with Col/haNPs

**Figure 5:**
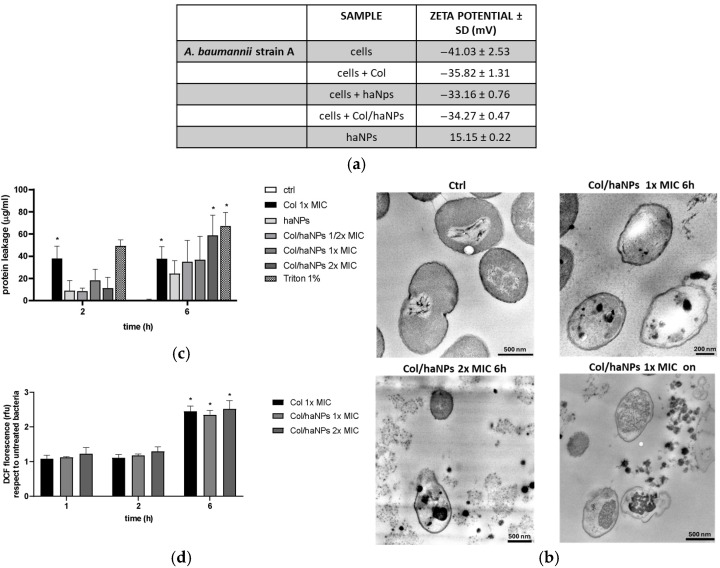
(**a**) Zeta-potential values recorded for bacterial cells stimulated for 1 h in the absence or in the presence of free Col, haNPs, and Col/haNPs (½ × MIC); zeta-potential values of haNPs in solution are also shown. Mean ± SD (mV) of 3 independent measurements (10 runs each) is presented. (**b**) Morphological analyses of *A. baumannii* cells by TEM. Representative images of untreated cells or bacteria after treatment with Col/haNPs at 1 × or 2 × MIC concentrations are shown (scale bars: 200 or 500 nm). (**c**) Effect of nanoparticles on protein leakage of bacterial cells. Bacteria were treated with free Col (1 × MIC), haNPs, or Col/haNPs (1 × and 2 × MIC) and evaluated for protein content (μg/mL). Triton 1% was used as a positive control. Mean ± SEM of 5 independent experiments * <0.05 versus untreated bacteria (ctrl) by Student’s *t*-test. (**d**) Effect of free Col (1 × MIC) or Col/haNPs (1 × and 2 × MIC) on bacterial ROS production, expressed as DCF fluorescence with respect to untreated bacteria. Mean ± SEM of 4 independent experiments. * <0.05 versus untreated bacteria (relative fluorescence intensity set as 1) by Student’s *t*-test.

To assess the effect of Col/haNPs on the bacterial cell surface, zeta potential measurements were performed. Untreated *A. baumannii* strain A displayed an average zeta potential of −41.03 ± 2.53 mV. Exposure to ½ × MIC of free Col (80 µg/mL) increased the zeta potential to −35.82 ± 1.31 mV, consistent with previous reports. Treatment with Col/haNPs (½ × MIC) or empty haNPs produced similar effects, shifting the potential to −33.16 ± 0.76 mV and −34.27 ± 0.47 mV, respectively. Empty haNPs in water exhibited a positive zeta potential of +15.15 ± 0.22 mV (Figure 5a). These findings indicate an electrostatic interaction between the positively charged chitosan-coated haNPs and the negatively charged bacterial surface, resulting in partial neutralization of the bacterial surface charge.

### 2.10. Morphological Alterations Induced by Col/haNPs

Transmission electron microscopy (TEM) was performed to evaluate morphological alterations of MDR *A. baumannii* after exposure to Col/haNPs and to capture different stages of nanoparticle–bacteria interaction and cellular damage. Untreated control cells showed normal morphology, with well-defined and intact cell walls and plasma membranes. In contrast, cells treated with 1 × MIC Col/haNPs for 6 h displayed accumulation of nanoparticles on the cell wall/membrane and within the cytoplasm, accompanied by structural alterations, ultimately leading to cell deformation. Prolonged exposure (1 × MIC for 18 h) or higher concentration (2 × MIC for 6 h) resulted in extensive lysis, loss of cytoplasmic content, and complete shrinkage of the cytoplasmic membrane (Figure 5b).

### 2.11. Effect of haNP/Col on Protein Leakage and Reactive Oxygen Species (ROS) Generation

To assess cytoplasmic protein leakage, supernatants were assayed by the Bradford method. At 2 h, significant release was detected only in cultures treated with free Col (Col 1 × MIC) or with 1% Triton X-100 (positive control). In contrast, haNPs alone and haNPs loaded with Col (Col/haNPs) at ½×, 1×, or 2 × MIC produced low, non-significant leakage, with no differences among nanoparticle groups. By 6 h, leakage increased markedly in the Col/haNPs 2 × MIC group, surpassing that observed with free Col (Figure 5c).

Finally, to evaluate whether the generation of ROS contributes to a Col/haNPs triggered effect in bacteria, a fluorescence probe with 2′,7′-dichlorodihydro-fluorescein diacetate (DCFH-DA) was used to monitor the levels of ROS in cells after exposure to Col 1 × MIC or Col/haNPs at 1× MIC and 2 × MIC for 1, 2, and 6 h. At 6 h, Col/haNPs 1 × MIC enhanced ROS generation, reaching levels similar to those induced by Col (Figure 5d). Thus, we believe that the antimicrobial ability of Col/haNPs involves the generation of intracellular ROS and substantial protein leakage attributable to membrane injury.

## 3. Discussion

Extensive research has explored nanotechnological strategies to counteract resistant bacterial infections [8]. In this context, we assessed the activity and investigated the potential mechanisms of Col-loaded human albumin nanoparticles (Col/haNPs) against a panel of Col-R clinical *A. baumannii* isolates that had been comprehensively characterized at both phenotypic and genotypic levels. Population analysis profiling revealed heteroresistance in nine of 16 isolates (56.25%), underscoring the high prevalence of resistant subpopulations within isolates that may appear susceptible or borderline by routine testing. Most strains belonged to the globally disseminated MDR lineage ST2, whereas isolate A was assigned to ST636, and isolate 1R to ST187, a single-locus variant of ST2 [11,37]. Col/haNPs consistently reduced colistin MICs across resistant isolates and markedly lowered MIC values in nearly all heteroresistant strains. A MIC-lowering effect was also observed in colistin-susceptible reference strains and clinical isolates, indicating that nanoparticle-mediated potentiation was not restricted to fully resistant backgrounds. Notably, MIC values reached levels that, for free Col, would fall within the EUCAST susceptibility breakpoint (≤2 mg/L), for which standardized criteria are currently available only for the free drug. Conversely, a minor MIC-lowering effect was reported only in 2R control resistant strain, exhibiting only a minor reduction in colistin MIC in the presence of Col/haNPs (from >160 to >40 µg/mL; <4-fold). To minimize bias arising from differences in genomic backgrounds, this different behavior has to be considered within strains with a close genomic relationship, i.e., vs. 1R control strain. The minor MIC-lowering effect is likely attributable to an increased electrostatic repulsion of the outer membrane toward colistin (8.87% vs. 3.7%), potentially driven by the R263H mutation in pmrB, as previously reported [11]. This substitution, located in the HisKA (Histidine Kinase A) domain, is known to constitutively activate the PmrAB regulatory system, promoting lipopolysaccharide modification and associated with high colistin MICs. In contrast, the L208F mutation observed in 1-R resides within the HAMP (Histidine kinases, Adenylyl cyclases, Methyl-accepting chemotaxis proteins, and Phosphatases) domain and has been associated with a lower colistin MIC and repulsion rate [38]. Notably, the presence of R263H in 2R likely enhances colistin repulsion, reduces drug accumulation, and limits interaction with Col/haNPs, consistent with the minimal treatment effect observed experimentally.

To contextualize the general MIC-lowering effect response in strains A–G, we delineated their underlying resistance mechanisms. Genomic and phenotypic analyses highlighted alterations affecting the PmrCAB–lipid A modification axis, whereas plasmid-mediated *mcr* genes were not identified. The absence of an LPS-deficient phenotype was supported by vancomycin susceptibility testing, Col E-test patterns, the lack of disruptive mutations in LpxACD, and largely comparable *lpx* transcription levels [39]. Multiple amino acid substitutions were identified predominantly in PmrB, a recurrent locus of variation in Col-resistant *A. baumannii* [39]. A138T and A226T co-occurred in strains F and G and were also detected individually in other isolates. Although these variants have been associated with elevated Col MICs [40], other studies indicate that recurrent PmrB substitutions may not be independently sufficient to confer resistance [41], supporting a context-dependent contributory role. Additional PmrB substitutions were detected in the Histidine Kinase A domain/Dimerization and Histidine phosphotransfer domain (HisKA/DHp region) (L261F, V268G, L274F) and histidine kinase, adenyl cyclase, methyl-accepting protein, and phosphatase linker HAMP, not previously described in association with Col resistance (Q126K). Strain A carried the previously reported R263C variant within the same domain [42]. Increased *pmrA*, *pmrB*, and *pmrC* expression was observed in most Col-R strains, particularly in isolates carrying A226T or A138T substitutions or multiple PmrB polymorphisms, consistent with reports linking these alterations to stable resistance phenotypes and constitutive PmrCAB activation [39,43,44]. MALDI-TOF confirmed phosphoethanolamine (pEtN) modification of lipid A, with strain-specific patterns of pEtN decoration. However, neither the extent of *pmrCAB* transcription nor the degree of lipid A modification strictly correlated with MIC values, indicating that lipid A remodeling represents a major but not exclusive determinant of Col resistance [39,45]. Variations in *galU*, a gene involved in the biosynthesis of UDP-glucose and surface glycans, were previously identified in strains 1–9 [11]. Extending this analysis to strains A–G, we confirmed a distribution consistent with that previously reported, with I245T predominating among heteroresistant isolates and I273V (often with Q140L) more frequently detected in fully resistant strains. Given that Col/haNPs displayed greater activity against heteroresistant isolates, differences in envelope architecture potentially linked to galU-dependent glycan variation may contribute to the differential response. However, the functional relevance of these substitutions remains to be established. The RND efflux system AdeABC, regulated by AdeRS, is frequently implicated in multidrug resistance. In our isolates, substitutions in AdeB (T626R and T669S) were observed among fully resistant strains, whereas the heteroresistant strain C carried a regulatory AdeS substitution (I62M) and displayed the highest *AdeB* expression. As AdeRS mutations have been linked to constitutive pump overexpression [46,47], I62M may contribute to the elevated *adeB* expression observed here. No significant variation or consistent overexpression was observed for *adeIJK*, supporting a more limited role for this system compared to AdeABC [48]. Mutations were detected in the regulator AdeN (N58T and E198*), previously reported in multidrug-resistant Acinetobacter [19,20]. In contrast, elevated *adeG* expression in the absence of variants in AdeG, and in the regulator AdeL, suggests additional regulatory complexity, consistent with prior evidence [49,50,51,52].

Qualitative EtBr cartwheel profiling indicated active efflux capacity across our strains, and the pronounced reduction in Col MIC following treatment with the proton motive force (PMF) uncoupler CCCP supports the involvement of PMF-dependent mechanisms, despite its broader effects on membrane physiology [53]. Empty haNPs combined with free Col markedly reduced Col MICs across all strains. Robust checkerboard assays confirmed strong synergy between Col and empty haNPs. Notably, this potentiation was selective for Col, as minimal effects were observed for ciprofloxacin and chloramphenicol under identical conditions. This selectivity indicates that potentiation depends on drug-specific physicochemical properties, including molecular structure and charge distribution, as well as envelope-associated pathways [54,55,56]. Albumin’s intrinsic ability to bind diverse molecules through electrostatic interactions may facilitate preferential Col–nanoparticle association and enhance local drug retention at the bacterial surface [8,31]. Membrane depolarization induced by the combination of Col and haNPs was comparable to that observed with CCCP treatment. Intracellular accumulation assays further showed that both CCCP and haNPs significantly increased intracellular Col levels when combined with the antibiotic. Together, these findings indicate that haNPs induce a CCCP-comparable functional phenotype [57] and are compatible with perturbation of membrane energetics, with possible downstream effects on PMF-dependent transport processes. However, the present data do not directly demonstrate specific efflux pump inhibition and do not exclude additional membrane/envelope-associated mechanisms.

Multiple independent analyses (zeta potential, DLS, FTIR, and TEM) supported direct haNP–cell surface interaction. Reduced nanoparticle zeta potential after free Col combination and partial neutralization of bacterial surface charge upon exposure are consistent with electrostatic association and enhanced envelope-level drug presentation. TEM confirmed extensive nanoparticle–cell interaction and progressive membrane disruption, while protein leakage and ROS generation at later time points suggest secondary membrane injury that may amplify bactericidal activity.

Overall, our data suggest that Col/haNPs affect membrane properties and intracellular colistin accumulation. However, these findings are not sufficient to directly demonstrate these mechanisms, and alternative explanations, including increased membrane permeability or altered envelope interactions, cannot be excluded. Further studies will be required to directly assess PMF components, efflux activity, and the reversibility of haNP-induced effects.

Col/haNPs exerted a particularly pronounced effect on heteroresistant isolates. Because heteroresistance reflects a threshold-dependent population structure in which resistant subpopulations coexist with more susceptible cells, modest increases in effective envelope-associated Col exposure may suppress the resistant fraction and lower overall MIC values [58,59]. Given that Col heteroresistance is frequently underrecognized in routine testing and rarely addressed in nanoparticle–antibiotic studies, the enhanced activity of Col/haNPs in this subgroup addresses a clinically relevant and underexplored gap. However, also haNPs combined with free Col showed activity on heteroresistant isolates.

Notably, the combination of free Col with haNPs showed a potentiating effect, which may be related to differences in the immediate availability of the drug compared with Col-loaded haNPs. However, it is important to consider that Col/haNPs release the drug in a sustained manner, with approximately 30% released after 24 h, as previously reported [10].

Importantly, given that CCCP is a toxic experimental uncoupler and not clinically applicable, the observation that haNPs reproduce a CCCP-like potentiating effect underscores their translational potential as a more viable strategy for Col enhancement. Furthermore, the ability of haNPs to potentiate Col both as a carrier formulation and upon co-administration with free drug provides a rationale for exploring novel therapeutic strategies, allowing Col dose reduction and a consequent decrease in drug toxic effects.

## 4. Materials and Methods

### 4.1. Bacterial Strains

A total of 16 clinical *A. baumannii* strains were analyzed, comprising both MDR and Col-resistant isolates. (Table 1). Seven Col-resistant XDR *A. baumannii* strains, designated A to G, were isolated from various clinical specimens (broncho/tracheal aspirates, bronchoalveolar lavage, blood, and rectal swab) collected from patients hospitalized in the Intensive Care Unit at the University Hospital “A.O.U. Città della Salute e della Scienza di Torino”. Additionally, nine previously characterized clinical control XDR *A. baumannii* strains, labeled 1–9R, obtained from bronchoalveolar lavage, blood, and wound samples, were provided by the MMAR Laboratory, University of Catania (Catania University Hospitals) [11]. Three Col-susceptible *A. baumannii* clinical strains (from MMAR Laboratory or “A.O.U. Città della Salute e della Scienza di Torino”), *A. baumannii* strains ATCC 17978 and ATCC 19606 (from American Type Culture Collection), and *A. baumannii* ACICU (kindly provided by Paolo Visca) were used as control strains. *E. coli* NCTC 13846 MCR-1 positive was used for screening of Col resistance and comparative studies. All isolates were unambiguously identified as *A. baumannii* by the Vitek^®^2 system (bioMérieux, Marcy l’Etoile, France) and MALDI-TOF MS (Bruker Daltonics, Bremen, Germany).

### 4.2. Preparation and Characterization of Col-Loaded Chitosan-Coated Albumin Nanoparticles (haNPs)

Blank and Col-loaded human albumin nanoparticles (haNPs and Col/haNPs) were prepared by a purposely tuned double-emulsion method, as reported in the patent (Italian Patent N° 102020000022984, University of Turin), and characterized as previously published [10]. In particular, the physico-chemical parameters, the morphology, the loading capacity, and the stability over time were determined.

### 4.3. Antimicrobial Susceptibility Testing

Antimicrobial susceptibility testing was performed using an NMDR panel in MicroScan WalkAway 96Plus (Beckman Coulter srl, Milan, Italy) according to the manufacturer’s instructions. The MICs of Col, free albumin, haNPs or Col/haNPs were determined by microdilution assay in cation-adjusted Mueller–Hinton broth (MHB II; Sigma-Aldrich, Saint Louis, MO, USA) according to the Clinical and Laboratory Standards Institute (CLSI) and the EUCAST guidelines. Free sulfate Col (Sigma-Aldrich), haNPs, and Col/haNPs were diluted in serial twofold dilutions (final concentrations from 160 to 0.078 μg/mL for free Col and 40 to 0.019 μg/mL for the loaded nanoparticles). Results were interpreted using EUCAST breakpoints (http://www.eucast.org/clinical_breakpoints/, last accession 12 January 2026) (≤2 mg/L for COL-S and >2 mg/L for COL-R). To verify bacterial cell viability and determine the MIC, 20 μL of a 1 mg/mL MTT stock solution was added to each well.

### 4.4. Population Analysis Profile (PAP) Assay

Col heteroresistance was determined by population analysis profiling (PAP) according to previously published protocols. Briefly, PAP assays were performed by plating 50 μL of a bacterial suspension (10^8^ colony-forming units (CFU)/mL) onto MHB II agar plates containing serial dilutions (2–128 mg/L) of Col sulfate. After incubation at 37 °C for 48 h, colonies were counted to determine the frequency of bacteria able to grow at each Col concentration relative to the Col-free control plate. Three biological replicates were performed. *A. baumannii* ATCC19606 was used as the control strain. Col heteroresistance was defined as the presence of detectable subpopulations able to grow at Col concentrations > 2 mg/L in isolates with a Col MIC ≤ 2 mg/L. Morphologically distinct subpopulations obtained from Col-containing agar plates, designated COL-R variants, were further evaluated for MIC values and stored as frozen stocks.

### 4.5. Whole Genome Sequencing (WGS)

DNA from the strains was extracted using the MasterPureTM Complete DNA and RNA Purification Kit following the producers’ instructions (LGC Biosearch Technologies, Inc., Middleton, WI, USA). A preliminary enzymatic lysis step was performed by using 25 µL of lysozyme [50 mg/mL] for each isolate. Whole Genome Shotgun sequencing was carried out by NOVOGENE (UK) using an Illumina NovaSeq platform (San Diego, CA, USA), leading to 2 × 150 bp reads. For whole bacterial genome analysis, the automated pipeline TORMES v2.0* with default parameters was used for quality filtering, assembly, and genome annotation [60].

### 4.6. Phylogeny and Genomic Epidemiology

Genomic relationships among the strains were investigated using the CSI Phylogeny tool. Genomic epidemiology analysis was performed using ResFinder (v4.1) and K-mer Resistance (v2.2) to identify acquired antimicrobial resistance (AMR) genes and known non-synonymous single nucleotide polymorphisms (nsSNPs) associated with AMR, applying thresholds of 98% nucleotide identity and a minimum coverage of 60%. Multilocus sequence typing (MLST) was determined in silico using the MLST software (v1.8) according to the Oxford and Pasteur Institute databases. Mobile genetic elements (MGEs) were identified using MobileElementFinder (v1.0.3), while prophage regions were detected using the PHAge Search Tool (PHAST), considering only prophages with a completeness score > 90. The presence of CRISPR/Cas systems and spacers in the analyzed genomes was assessed using CRISPRFinder. CRISPR array types were further evaluated using CRISPRCasdb, where CRISPR4 corresponds to level 4 CRISPRs (the most reliable), whereas levels 1–3 may represent false CRISPRs. Bacterial surface polysaccharide loci were investigated using Kaptive, which identifies the outer-core (OC) locus of the lipooligosaccharide (LOS), forming the lipopolysaccharide (LPS), and the K locus (KL) involved in capsular polysaccharide (CPS) synthesis [11].

### 4.7. Single Nucleotide Polymorphisms (SNPs)

SNP calls were carried out from the PE library raw reads as already published [38] on *A. baumannii* ACICU RefGen mapping.

### 4.8. Expression Analysis of Col Resistance Associated Genes

Starting from a single colony, bacterial cells of *A. baumannii* COL-R strains (A–G) and *A. baumannii* ATCC19606, were subcultured overnight (logarithmic phase) with shaking at 100 rpm. Total RNA was isolated using AFTSpin Bacterial Fast RNA Extraction Kit (ABclonal, Düsseldorf, Germany) and treated with PureLinkTM DNase (Invitrogen by Thermo Fisher Scientific, Waltham, MA, USA).

In parallel, an overnight culture of *A. baumannii* strains was subcultured 1:100 in fresh MHB II and grown at 37 °C with shaking for 6h, then cells were stimulated with a sub-MIC dose (½×) of Col/haNPs, haNPs, and free Col (at the same dose of Col/haNPs) overnight with shaking at 37 °C. RNA was extracted as reported above. cDNA was synthesized using iScriptTM cDNA Synthesis Kit (Biorad, Hercules, CA, USA), and Real-time PCR performed with CFX96 Touch Real-Time PCR Detection System (Biorad) using SsoAdvancedTM Universal SYBR^®^ Green Supermix Kit (Biorad). The reaction condition was set as a one-step method as follows: initial denaturation at 95 °C for 2 min, 40 cycles consisting of denaturation at 95 °C for 5 s, annealing at 60 °C for 30 s. Reactions were run in duplicate. The expression of the COL-R associated genes was assessed using specific primers as listed in the table (Table S4). rpoB was used as reference gene to normalize expression levels. Data were calculated using the comparative CT method and expressed as the mean ± SEM.

### 4.9. Colistin Dependence Linked to LPS Loss

Col resistance via lipopolysaccharide loss results in partial Col dependence. Visualization of Col dependence was performed by E-test^®^ (bioMérieux, Marcy-l’Étoile, France) as previously described [23]. Briefly, bacterial strains were inoculated onto MHA plates using a sterile cotton swab after adjusting a bacterial suspension in physiological saline to 0.5 McFarland. E-test strips were then applied to the agar surface, and the plates incubated at 37 °C for 24 h before examination. Col dependence was defined by the observation that Col-R LPS-deficient derivatives grow more abundantly in the Col-containing zones adjacent to the E-test strip than in Col-free areas of the plate [23].

### 4.10. Susceptibility to Vancomycin

Vancomycin susceptibility was assessed using the broth microdilution method in order to evaluate the loss of LPS in Col-resistant *A. baumannii* isolates, as vancomycin displays markedly increased activity against LPS-deficient strains.

### 4.11. CPD-E Test

The CDP-E test is a validated method able to differentiate plasmid-mediated Col resistance from resistance mediated by chromosomal mutations. The assay was performed as previously reported by Yauri Condor K et al. [24]. Col disks were placed on Mueller–Hinton agar (MHA) plates for 2 h, removed, and plates left for 24 h. A bacterial suspension of each tested strain at 0.5 McFarland was spread on plates, then a disk of EDTA at 1 μM was added where the Col disk was previously located, and another EDTA disk placed to verify no inhibitory effect of EDTA. Diameters of inhibition zones around Col and EDTA disks were measured after 18 h of incubation. 

### 4.12. Lipid A Extraction and MALDI-TOF MS Characterization

Lipid A from *A. baumannii* was extracted using the MBT Lipid Xtract™ Kit according to the manufacturer’s instructions (Bruker Daltonics, Bremen, Germany). Briefly, bacteria were grown overnight on MHA plates. The equivalent of a 1 µL inoculation loop was transferred into a 1.5 mL low-binding microtube and mixed with 50 µL of MBT Lipid Xtract Hydrolysis buffer. Subsequently, 44 µL of the suspension was discarded, and the remaining 6 µL was heated at 90 °C for 10 min with the tube lid closed. The tubes were then left open for 2 min to allow the buffer to fully evaporate. The dried pellets were washed with 50 µL of MBT Lipid Xtract washing buffer for a few seconds without dissolving the pellet, after which the washing buffer was completely removed by pipetting. Finally, 5 µL of matrix solution was pipetted to resuspend the dried pellet, and 2 µL of the suspension was spotted onto an MSP 96 polished steel target (Bruker Daltonics). After air-drying, lipid A structural spectra were acquired using the MALDI Biotyper sirius^®^ system (Bruker Daltonics) in negative ion mode. Spectra were recorded in linear negative-ion mode (laser intensity 30–40%, ion source 1 = 15.00 kV). Each spectrum corresponded to the accumulation of 200–1000 laser shots randomly distributed across the spot. After spectral acquisition, data were manually evaluated using FlexAnalysis v4.0 software (Bruker Daltonics). The overall MALDI-TOF MS analysis range was *m*/*z* 400–3200, while lipid A mass analysis was focused on the range *m*/*z* 1300–2300 [61].

### 4.13. (EtBr)-Agar Cartwheel Method

To evaluate the expression of efflux pumps in *A. baumannii* strains, a previously reported agar-based method was performed. Bacterial strains were grown in broth overnight at 37 °C. The OD of the cultures was adjusted with PBS to 0.5 of a McFarland standard. Tryptic Soy Agar (TSA; Sigma-Aldrich, Saint Louis, MO, USA) plates containing EtBr concentrations ranging from 0 to 2 mg/L were prepared on the same day of the experiment and protected from light. The plates were then divided into 8 sectors by radial lines (cartwheel pattern) as exemplified in Figure 3b Cultures were swabbed on EtBr-agar plates. The plates included the reference strain *A. baumannii* ATCC19606, used as a comparative control, *E. coli* NCTC, and the *A. baumannii* COL-R clinical strains. The EtBr-agar plates were then incubated at 37 °C overnight and examined under a UV transilluminator (Benchmark Accuris™, Sayreville, NJ, USA). The MCEtBr that produced fluorescence of the bacterial mass was recorded. An index for efflux activity of the MDR strains was calculated as previously reported by Martins M et al. [62].

### 4.14. Effect of CCCP and haNPs on Col MIC Using Microdilution Method: Efflux Pump Inhibitor (EPI)-Based Microplate Assay

The susceptibility to Col was evaluated in the presence and absence of the inhibitor CCCP (Sigma–Aldrich, St Louis, MO, USA). Col with a concentration ranging from 160/80 to 0.078/0.039 μg/mL was added to each plate containing MHB II. In the corresponding plate, CCCP was present at a final concentration of 10 μg/mL, as previously reported [5,27]. Control wells with CCCP alone were added to exclude CCCP toxicity. MIC fold change (reduction) was calculated as the ratio of the CCCP-free Col MIC level to that of the CCCP-added Col. A 4-fold or greater decrease in the MIC values after the addition of CCCP was considered as a criterion of significance, as previously proposed [5,27]. In parallel, to demonstrate a possible role of blank haNPs as EPI, the same were added (8–16 μM albumin concentration) instead of CCCP. The effect of CCCP and haNPs on other antibiotics, ciprofloxacin (Cpx) and chloramphenicol (Caf), was also evaluated.

### 4.15. Synergistic Interaction Studies

The synergistic interaction between Col and haNPs was evaluated using the checkerboard assay as previously described [33]. Col and haNPs were prepared in two-fold serial dilutions and combined to generate different concentration combinations, with Col along the x-axis and haNPs along the y-axis. A final bacterial suspension of 1 × 10^5^ CFU/mL was added to each well. After incubation at 37 °C for 18 h, bacterial viability was assessed using the MTT assay. The fractional inhibitory concentration index (FICI) was calculated as the sum of the MIC of each compound in combination divided by the MIC of the compound tested alone, according to the following formula:

FICI = (MIC_AB_/MIC_A_) + (MIC_BA_/MIC_B_)

where MIC_AB_ is the MIC of drug A in combination, MIC_A_ is the MIC of drug A alone, MIC_BA_ is the MIC of drug B in combination, and MIC_B_ is the MIC of drug B alone. FICI values were interpreted as follows: FICI ≤ 0.5, synergy; 0.5 < FICI ≤ 1, additive effect; 1 < FICI ≤ 4, indifferent effect; and FICI > 4, antagonism. Each experiment was performed in triplicate.

### 4.16. Bacterial Membrane Potential Assay

Briefly, subcultures of *A. baumannii* were made from an overnight culture and grown to the mid-log phase. Cells were incubated with Col/haNPs (1 × MIC), haNPs (at the same dose of Col/haNPs), free Col (1 × MIC), free Col in combination with haNPs (at the same dose of Col/haNPs)at 37 °C for 1.5 h without shaking. CCCP (100 μg/mL) was used as a positive control of effective membrane depolarization, as previously reported [63]. Then, the cells were washed and resuspended with phosphate-buffered saline (PBS, pH 7.4) and diluted to 0.5 McFarland. The BacLightTM Bacterial Membrane Potential Kit (Invitrogen, Carlsbad, CA, USA) was used to measure the bacterial membrane potential. Stained bacterial cells were evaluated using a FACSCanto II flow cytometer (Beckton Dickinson, Franklin Lakes, NJ, USA) to collect the red and green mean fluorescence intensity (MFI).

### 4.17. Col Accumulation Studies

Intracellular accumulation of Col in response to different treatments with Col (1 × MIC), Col + haNPs (16 μM albumin concentration), Col + CCCP (10 μg/mL), or Col/haNPs (1 × MIC) was evaluated using HPLC assay. An overnight culture of bacteria was subcultured 1:100 in fresh MHB II and grown at 37 °C to the mid-log phase before treatment. Bacteria were stimulated for 3 h at 37 °C with shaking.

After incubation the bacteria were pelleted at 4000 rpm for 5 min, and the supernatant was discarded. The pellets were re-suspended in PBS twice, pelleted at 12,000 rpm for 2 min, and aliquoted into 1.5 mL tubes. To lyse the samples, each pellet was resuspended in 200 μL of ultrapure water and subjected to three freeze–thaw cycles consisting of 3 min in liquid nitrogen followed by 3 min in a water bath at 65 °C. The lysate was then centrifuged at 12,000 rpm for 2 min at room temperature, and the supernatant was collected. The remaining debris was resuspended in 100 μL of methanol and centrifuged again under the same conditions. The resulting supernatant was combined with the previously collected supernatant. Finally, residual debris was removed by centrifugation at 14,600 rpm for 10 min at room temperature. Supernatants were analyzed by UPLC–MS/MS for determination of Col-A and Col-B. UPLC-MS/MS analysis of the two analytes was performed using a Waters Acquity TQD system with a Waters (Milford, MA, USA) BEH C18 column (2.1 × 50 mm, 1.7 µm) thermostated at 40 °C. Elution was performed at a flow rate of 0.4 mL/min using a gradient of 0.1% formic acid in water (water-FA) to 0.1% formic acid in acetonitrile (ACN-FA). The adopted gradient profile is as follows: (% ACN-FA, min): 10, 0; 10, 1.30; 30, 3.90; 51, 5.20; 100, 5.60; 100, 7.40. The two species were detected in ESI + MRM, with a capillary voltage of 3.5 kV, using the following fragmentations: *m*/*z* 585.5 -> 101.0 (collision energy 20 eV, COL-A quantification), *m*/*z* 585.5 -> 576.00 (collision energy 20 eV, COL-A qualification), *m*/*z* 579.0 -> 101.0 (collision energy 24 eV, COL-B quantification), and *m*/*z* 579.0 -> 570.00 (collision energy 20 eV, COL-B qualification). For COL-A, a linearity range of 2.35 to 23.5 ppm was obtained, with a standard deviation of 0.07 ppm at 2.35 ppm. The LOD and LOQ were determined to be 1.57 ppm and 2.35 ppm, respectively. For COL-B, a linearity range of 3.25 to 6.5 ppm was verified, with a standard deviation of 0.07 ppm at 3.25 ppm. The LOD and LOQ were both found to be 3.25 ppm.

The number of CFUs was determined for each experimental setting to relate Col accumulation to bacterial count.

### 4.18. Zeta Potential Measurement

Changes in the surface charge of *A. baumannii* COL-R strains to examine the effect and interaction of haNPs with the COL-R strain A of *A. baumannii* were investigated. Bacteria were prepared at mid-log growth phase and stimulated with free Col, haNPs, and Col/haNPs (½ × MIC) for 1 h in shaking conditions (37 °C, 100 rpm) and then prepared for zeta potential measurements. Samples were adequately cleansed, washing the bacterial suspension twice with Milli-Q™ water. Washed cells, resuspended in Milli-Q™ water at a concentration of ~1 × 10^8^ CFU/mL, were further diluted (5-fold dilution) immediately before measurement. A zeta potential analyzer (Zetasizer Nano ZS™, Malvern Instruments Ltd., Malvern, UK) at 150 V was used to measure the electrophoretic mobility (EPM) of bacterial cells, subsequently converted to zeta potential values according to the Helmholtz–Smoluchowski theory. Measurements were performed at 25 °C in Milli-Q™ water and are reported as mean ± SD (mV) from three independent samples prepared on separate days, each measured in triplicate with 10 runs per measurement. Electrode polarization was performed before analysis under identical conditions. Between measurements, electrodes were rinsed with ethanol and Milli-Q™ water.

### 4.19. FTIR Analysis

Fourier-transform infrared spectroscopy (FTIR) analysis was performed using a Perkin Elmer Spectrum Spotlight 100 FTIR spectrophotometer equipped with Spectrum 10 MultiSearch Module software (PerkinElmer, Waltham, MA, USA). The analyses were carried out using a versatile attenuated total reflectance (FTIR-ATR) sampling accessory equipped with a diamond crystal plate. The FTIR spectra of Col, haNPs, Col/haNPs, and haNPs + free Col were collected in the spectral range of 4000–650 cm^−1^.

### 4.20. Ultrastructure of the Bacteria

For TEM observation bacteria were cocultured with or without Col/haNPs for 6 h or overnight, and bacterial pellets fixed in a solution of 1.25% glutaraldehyde (Sigma-Aldrich, Saint Louis, MO, USA), 1% Paraformaldehyde, and 0.5% sucrose (Merck, Darmstadt, Germany) in 0.1 M Sorensen phosphate buffer 7.4, for 6–8 h, then washed and stored in 0.1M Sorensen phosphate buffer with addition of 1.5% sucrose at 4 °C.

Successively, the samples were immersed at 4 °C for 2 h in 2% osmium tetroxide (Electron Microscopy Science) in the same buffer solution. The cells were then dehydrated via an ethanol series (50%, 70%, 80%, 90%, and 100%, respectively) and embedded in Epon/Araldite resin (polymerization at 60 °C for 48 h) after two passages in propylene oxide (Sigma Aldrich).

Thin sections of the samples were cut in a thickness range of 50–70 nm with an ultramicrotome (Ultracut UCT, Leica Microsystems, Wetzlar, Germany). Sections were collected and placed on grids previously coated with pioloform film and stained with a solution of 4% UAR-EMS uranyl acetate replacement in distilled water, and analyzed using a JEM-1010 transmission electron microscope (JEOL, Tokyo, Japan) equipped with a Megaview-III digital camera and a Soft-Imaging-System (SIS, Münster, Germany) for the computerized acquisition of the images.

### 4.21. Protein Leakage Assay (Membrane Integrity Assay)

An overnight culture of *A. baumannii* was subcultured at 4 × 105 CFU/mL and incubated in the absence or presence of ½ ×, 1 ×, and 2 × MIC of Col/haNPs, free Col at 1 × MIC, haNPs (at the same dose of Col/haNPs), or Triton 1% as positive control for 2 and 6 h at 37 °C in PBS with shaking and subsequently pelleted at 12,000 rpm for 20 min. The supernatant was collected, and the leakage of proteins measured using a NanoDrop One spectrophotometer (Thermo Fisher Scientific) to record the absorbance at 562 nm. A BCA Protein Assay kit (EMD Millipore Corp., Burlington, MA, USA) was used to create a standard curve and quantify proteins.

### 4.22. Reactive Oxygen Species (ROS) Measurement

The production of reactive oxygen species (ROS) in bacteria following haNP treatment was measured using the DCFDA assay as previously described [64]. Overnight bacterial cultures were subcultured as described above for the protein leakage assay. Cells were then treated with free Col (1 × MIC) or Col/haNPs (1 × and 2 × MIC) for 1, 2, and 6 h at 37 °C in black 96-well microplates. Subsequently, 5 µM of 2′,7′-dichlorofluorescin diacetate (DCFDA) was added to the cell suspension and incubated at 37 °C for 30 min in the dark. Fluorescence was measured using a multimode microplate reader Victor (Revvity, Waltham, MA, USA) with excitation and emission wavelengths of 485 and 535 nm, respectively. Relative fluorescence intensity was calculated after background subtraction and normalized to the fluorescence of untreated cells.

### 4.23. Statistical Analysis

All experiments were conducted in at least three independent experiments. Student’s *t* test was used, and a *p*-value ≤ 0.05 was considered statistically significant. Results were presented as mean ± SEM, except for zeta potential measurements presented as mean ± SD. All data were analyzed using GraphPad PRISM 8.0 software.

### 4.24. Patents

Italian Patent N° 102020000022984 (University of Turin, Italy): “Composizione comprendente nanoparticelle di albumina incapsulanti antibiotici”.

## Figures and Tables

**Table 1:** Source, genomic characterization, and epidemiology of clinical COL-R *A. baumannii* (*Ab*) strains.

Strains	Hospital Wards	Source	gPhyl Cluster	MLST Pasteur	KL/OCL Type	Resistome	
						b-lactams	AGs
*Ab* A (F-R)	ICU	BAL	IV	ST-638	KL40/OCL2	*blaADC-25* *blaOXA-66* *blaOXA-72*	*ant(3″)-Ia* *aac(3)-Ia* *aph(3′)-Ia* *aph(3′)-VIa*
*Ab* B (F-R)	ICU	Trachealaspirate	III	ST-2	KL22/OCL3	*blaOXA-23* *blaADC-25* *blaOXA-82*	*aph(3′)-Via* *aadA2* *ant(2″)-Ia*
*Ab* C (H-R)	ICU	Blood	II	ST-2	KL7/OCL1d	*blaOXA-23* *blaADC-25* *blaOXA-66*	*aph(3′)-Ia* *aph(3″)-Ib* *aph(6)-Id* *ant(3″)-Ia*
*Ab* D (F-R)	ICU	BAL	III	ST-2	KL22/OCL3	*blaOXA-23* *blaADC-25* *blaOXA-82*	*aph(3′)-Via* *aadA2* *ant(2″)-Ia*
*Ab* E (F-R)	ICU	Anal swab	III	ST-2	KL22/OCL3	*blaOXA-23* *blaADC-25* *blaOXA-82*	*aph(3′)-Via* *aadA2* *ant(2″)-Ia*
*Ab* F (H-R)	ICU	Blood	II	ST-2	KL2/OCL1c	*blaOXA-23* *blaADC-25* *blaOXA-66*	*aph(3″)-Ib* *aph(6)-Id* *armA*
*Ab* G (H-R)	ICU	Bronchoaspirate	II	ST-2	KL2/OCL1c	*blaOXA-23* *blaADC-25* *blaOXA-66*	*aph(3″)-Ib* *aph(6)-Id* *armA*
*Ab* 1R (F-R)	ICU	BAL	III	ST-187	KL22/OCL3	*blaOXA-23* *blaADC-25* *blaOXA-82*	*aadA2* *ant(2″)-Ia* *aph(3′)-VIa*
*Ab* 2R (F-R)	ICU	BAL	III	ST-2	KL22/OCL3	*blaOXA-23* *blaADC-25* *blaOXA-82*	*aac(3)-Ia* *aadA1* *aadA2* *ant(2″)-Ia* *aph(3′)-VIa*
*Ab* 3R (F-R)	ICU	BAL	II	ST-2	KL3/OCL1	*blaOXA-23* *blaADC-25* *blaOXA-66* *blaTEM-1D*	*aph(3″)-Ib* *aph(3′)-Ia* *aph(6)-Id* *armA*
*Ab* 4R (H-R)	ICU	BAL	II	ST-2	KL28/OCL1	*blaOXA-23* *blaADC-25* *blaOXA-66*	*aph(3″)-Ib* *aph(6)-Id*
*Ab* 5R (H-R)	ICU	BAL	I	ST-2	KL9/OCL1	*blaADC-25* *blaOXA-66* *blaOXA-72*	*aac(6′)-Ip* *aph(3″)-Ib* *aph(6)-Id* *armA*
*Ab* 6R (H-R)	ICU	BAL	I	ST-2	KL9/OCL1	*blaADC-25* *blaOXA-66* *blaOXA-72*	*aac(6′)-Ip* *aph(3″)-Ib* *aph(6)-Id* *armA*
*Ab* 7R (H-R)	BURN U	Blood	I	ST-2	KL9/OCL1	*blaADC-25* *blaOXA-66* *blaOXA-72*	*aph(3″)-Ib* *aph(6)-Id* *armA*
*Ab* 8R (H-R)	ICU	Blood	I	ST-2	KL9/OCL1	*blaADC-25* *blaOXA-66* *blaOXA-72*	*aph(3″)-Ib* *aph(6)-Id* *armA*
*Ab* 9R (H-R)	TRAUMA U	Wound	I	ST-2	KL9/OCL1	*blaADC-25* *blaOXA-66* *blaOXA-72*	*aph(3″)-Ib* *aph(6)-Id* *armA*

**Table 2:** Col susceptibility and antimicrobial activity of Col/haNPs using broth microdilution assay of the clinical COL-R, COL-S, and *A. baumannii* (*Ab*) reference strains.

Strains	Col MIC (µg/mL)	Col/haNPs MIC (µg/mL)
*Ab* A (F-R)	>160	10
*Ab* B (F-R)	>160	5
*Ab* C (H-R)	1.25 (1.25–40)	0.078
*Ab* D (F-R)	>160	20
*Ab* E (F-R)	160	20
*Ab* F (H-R)	40 (10–160)	0.62
*Ab* G (H-R)	160 (20–160)	2.5
*Ab* 1R (F-R)	40	2.5
*Ab* 2R (F-R)	>160	>40
*Ab* 3R (F-R)	>160	20
*Ab* 4R (H-R)	1.25 (1.25–64)	0.078
*Ab* 5R (H-R)	40 (2–40)	1.25
*Ab* 6R (H-R)	40 (2–128)	1.25
*Ab* 7R (H-R)	80 (2–128)	1.25
*Ab* 8R (H-R)	2.5 (2–32)	0.16
*Ab* 9R (H-R)	1.25 (1–128)	0.31
*Ab* 1S	0.62	0.156
*Ab* 2S	0.31	0.039
*Ab* 3S	0.62	0.156
*Ab* ATCC19606	0.62	0.078
*Ab* ATCC17978	0.62	0.078
*Ab* ACICU	0.62	0.078

**Table 3:** MIC values of Col against the COL-R *A. baumannii* strains and *A. baumannii* ATCC19606 with and without the addition of CCCP (10 μg/mL) or haNPs (8–16 μM albumin concentration) were evaluated. A 4-fold or greater decrease in the MIC values after addition of CCCP or haNPs was considered a criterion of significance.

Strains	MIC Col (µg/mL)	MIC Col + CCCP (µg/mL)	Fold MIC Reduction (≥4×)	MIC Col + haNPs (µg/mL)	Fold MIC Reduction (≥4×)
*Ab* A	>160	5	**>32**	5	**>32**
*Ab* B	160	<0.156	**>1026**	2.5	**64**
*Ab* C	1.25	<0.156	**>8**	0.039	**32**
*Ab* D	160	<0.156	**>1026**	10	**16**
*Ab* E	160	<0.156	**>1026**	40	**4**
*Ab* F	80	1.25	**64**	0.156	**512**
*Ab* G	160	2.5	**64**	0.312	**512**
*Ab* ATCC 19606	0.625	0.078	**8**	0.039	**16**

## Data Availability

Raw reads have been deposited in the NCBI under the bioproject PRJNA1437057. The other data presented in this study are available on request from the corresponding author.

## Supplementary Materials

The following supporting information can be downloaded at: https://www.mdpi.com/article/10.3390/antibiotics15040410/s1, Figure S1: Phylogenetic tree of all clinical *A. baumannii* strains; Figure S2: Representative spectra of *A. baumannii* COL-R strain A and *A. baumannii* ATCC19606; Figure S3: FTIR spectra of Col, haNPs, and haNPs + Col; Table S1: Resistome profile of *A. baumannii* strains; Table S2: Evaluation of MIC of chloramphenicol (Caf) or ciprofloxacin (Cpx) in the presence of haNPs or CCCP; Table S3: MIC, change in MIC and FICI values of Col and haNPs of strains B and G with the checkerboard assay; Table S4: List of specific primers used to assess COL-R associated genes.

## Abbreviations

The following abbreviations are used in this manuscript:

ACN-FA	Formic acid in acetonitrile
AMR	Antimicrobial resistance
Caf	Chloramphenicol
CCCP	Carbonyl cyanide 3-chlorophenylhydrazon
CFUs	Colony forming units
CLSI	Clinical and Laboratory Standards Institute
Col	Colistin
Col/haNPs	Colistin-loaded albumin nanoparticles
Cpx	Ciprofloxacin
DiOC_2_(3)	3,3′-Diethyloxacarbocyanine, iodide
DLS	Dynamic Light Scattering
EPIs	Efflux pump inhibitors
EtBr	Ethidium bromide
EUCAST	European Committee on Antimicrobial Susceptibility Testing
FA	Formic acid
FICI	Fractional inhibitory concentration index
FTIR	Fourier Transform Infrared spectroscopy
GalN	galactosamine
gPhyl	Genomic phylogeny
haNPs	Human albumin nanoparticles
HPLC	High Performance Liquid Chromatography
ICUs	Intensive care units
LOS	Lipooligosaccharide
LPS	Lipopolysaccharide
MALDI-TOF MS	Matrix-Assisted Laser Desorption/Ionization Time-of-Flight Mass Spectrometry
MATE	Multidrug and toxic compound extrusion
MCEtBr	Minimum concentration of EtBr
MDR	Multidrug-resistant
MFS	Major facilitator superfamily
MGEs	Mobile genetic elements
MHA	Muller-Hinton agar
MHB II	Muller-Hinton broth
MI	Moderate impact
MIC	Minimum inhibitory concentration
MLST	Multi-Locus Sequence Typing
nsSNPs	Non-synonymous single nucleotide polymorphisms
OD	Optical density
PAP	Population analysis profiling
PEtN	Phosphoethanolamine
PHAST	PHAge Search Tool
PMF	Proton motive force
RND	Resistance-nodulation-division
ROS	Reactive Oxygen Species
SNPs	Single nucleotide polymorphisms
TEM	Transmission electron microscopy
TSA	Tryptic Soy Agar
UPLC-MS/MS	Ultra-Performance Liquid Chromatography-Tandem Mass Spectrometry
WGS	Whole-genome sequencing
XDR	Extensively drug-resistant
