# Supplementary material for: Albumin Nanoparticles Improve Colistin Performance Against Hetero- and Full-Resistant Clinical A. baumannii: A Mechanistic Study
Source: Antibiotics (Basel). 2026 Apr 17;15(4):410. doi: 10.3390/antibiotics15040410 (PMC13114173; doi:10.3390/antibiotics15040410)
Supplement: Supplementary file 1 [file antibiotics-15-00410-s001.zip › antibiotics-4237135-supplementary.pdf]

Table S1. Resistome profile and MGE (Mobile Genetic Elements).

STRAIN	MGE	ACQUIRED RESISTOME RESFINDER	RESISTANCE	RESISTOME CARD	RESISTANCE
**A**	IS17<br>Tn6018<br>IS6100<br>ISAba1<br>ISAba125<br>ISAba31<br>Composite transposon cn_1201_IS6100	blaADC-25<br>blaOXA-66<br>blaOXA-72<br>catA1<br>ant(3'')-Ia<br>aac(3)-Ia<br>sul1<br>aph(3')-Ia<br><br>aph(3')-VIa	unknown beta-lactam<br>unknown beta-lactam<br>unknown beta-lactam<br>Chloramphenicol<br>Streptomycin<br>Gentamicin<br>Sulfamethoxazole<br>kanamycin, paromomycin, neomycin, lividomycin, ribostamycin<br>Amikacin	adeC<br>adeB<br>adeA<br>adeR<br>adeS<br>A.baumannii_AmvA<br>A.baumannii_AbaQ<br>abeM<br>ADC-74<br>A.baumannii_AbaF<br>adeK<br>adeJ<br>adeI<br>OXA-66<br>adeN<br>adeH<br>adeF<br>adeG<br>adeL<br>abeS<br>OXA-72<br>catI<br>ANT(3'')-IIa<br>AAC(3)-Ia<br>sul1<br>APH(3')-Ia<br>APH(3')-VIa	glycylcycline;tetracycline<br>glycylcycline;tetracycline<br>glycylcycline;tetracycline<br>glycylcycline;tetracycline<br>glycylcycline;tetracycline<br>acridine_dye;macrolide<br>fluoroquinolone<br>acridine_dye;fluoroquinolone;triclosan<br>cephalosporin<br>fosfomycin<br>carbapenem;cephalosporin;fluoroquinolone;lincosamide;macrolide;penem;phenicol;rifamycin;tetracycl<br>carbapenem;cephalosporin;fluoroquinolone;lincosamide;macrolide;penem;phenicol;rifamycin;tetracycl<br>carbapenem;cephalosporin;fluoroquinolone;lincosamide;macrolide;penem;phenicol;rifamycin;tetracycl<br>cephalosporin;carbappenam<br>carbapenem;cephalosporin;fluoroquinolone;lincosamide;macrolide;penem;phenicol;rifamycin;tetracycl<br>fluoroquinolone;tetracycline<br>fluoroquinolone;tetracycline<br>fluoroquinolone;tetracycline<br>fluoroquinolone;tetracycline<br>aminocoumarin;macrolide<br>cephalosporin;penam<br>phenicol<br>aminoglycoside<br>aminoglycoside<br>sulfonamide<br>aminoglycoside<br>aminoglycoside
**B**	IS6100<br>ISAba125<br>ISAba13<br>IS26	blaADC-25<br>blaOXA-82<br>aadA2<br>ant(2'')-Ia<br>blaOXA-23<br>sul1<br>aph(3')-VIa	unknown beta-lactam<br>Imipenem<br>Streptomycin<br>Gentamicin;Tobramycin<br>Imipenem;Meropenem<br>Sulfamethoxazole<br>Amikacin	adeN<br>A.baumannii_AbaQ<br>abeM<br>A.baumannii_AbaF<br>adeC<br>adeB<br>adeA<br>adeR<br>adeS<br>A.baumannii_AmvA<br>ADC-25<br>OXA-115<br>adeH<br>adeF<br>adeG<br>adeL<br>abeS<br>aadA2<br>ANT(2'')-Ia<br>OXA-23<br>sul1<br>APH(3')-VIa<br>adeK<br>adeJ<br>adeI	carbapenem;cephalosporin; fluoroquinolone;lincosamide;macrolide;penem;phenicol;rifamycin;tetracycl<br>fluoroquinolone<br>acridine_dye;fluoroquinolone;triclosan<br>fosfomycin<br>glycylcycline;tetracycline<br>glycylcycline;tetracycline<br>glycylcycline;tetracycline<br>glycylcycline;tetracycline<br>glycylcycline;tetracycline<br>acridine_dye;macrolide<br>cephalosporin<br>cephalosporin;penam<br>fluoroquinolone;tetracycline<br>fluoroquinolone;tetracycline<br>fluoroquinolone;tetracycline<br>fluoroquinolone;tetracycline<br>aminocoumarin;macrolide<br>aminoglycoside<br>cephalosporin;penam<br>sulfonamide<br>aminoglycosid<br>carbapenem;cephalosporin;fluoroquinolone;lincosamide;macrolide;penem;phenicol;rifamycin;tetracycl<br>carbapenem;cephalosporin;fluoroquinolone;lincosamide;macrolide;penem;phenicol;rifamycin;tetracycl<br>carbapenem;cephalosporin;fluoroquinolone;lincosamide;macrolide;penem;phenicol;rifamycin;tetracycl
**C**	ISVsa3	tet(B)<br>aph(6)-Id<br>aph(3'')-Ib<br>sul1<br>ant(3'')-Ia<br>catB8<br>aac(6')-Ib<br>blaOXA-66<br>blaADC-25<br>blaOXA-23<br>aph(3')-Ia	Doxycycline;Tetracycline;Minocycline<br>Streptomycin<br>Streptomycin<br>Sulfamethoxazole<br>Streptomycin<br>Chloramphenicol<br>Amikacin;Tobramycin<br>unknown beta-lactam<br>unknown beta-lactam<br>Imipenem;Meropenem	abeS<br>adeL<br>adeG<br>adeF<br>adeH<br>A.baumannii_AbaQ<br>adeK<br>adeJ<br>adeI<br>A.baumannii_AbaF<br>abeM<br>tet(B)<br>APH(6)-Id<br>APH(3'')-Ib<br>adeR<br>adeA<br>adeB<br>adeC<br>sul1<br>ANT(3'')-IIa<br>catB8<br>AAC(6')-Ib7<br>OXA-66<br>ADC-30<br>OXA-23<br>APH(3')-Ia<br>A._baumannii_AmvA<br>adeN	aminocoumarin;macrolide<br>fluoroquinolone;tetracycline<br>fluoroquinolone;tetracycline<br>fluoroquinolone;tetracycline<br>fluoroquinolone;tetracycline<br>fluoroquinolone<br>carbapenem;cephalosporin;fluoroquinolone;lincosamide;macrolide;penem;phenicol;rifamycin;tetracycl<br>carbapenem;cephalosporin;fluoroquinolone;lincosamide;macrolide;penem;phenicol;rifamycin;tetracycl<br>carbapenem;cephalosporin;fluoroquinolone;lincosamide;macrolide;penem;phenicol;rifamycin;tetracycl<br>fosfomycin<br>acridine_dye;fluoroquinolone;triclosan<br>tetracycline<br>aminoglycoside<br>aminoglycoside<br>glycylcycline;tetracycline<br>glycylcycline;tetracycline<br>glycylcycline;tetracycline<br>glycylcycline;tetracycline<br>sulfonamide<br>aminoglycoside<br>phenicol<br>aminoglycoside<br>cephalosporin;penam<br>cephalosporin<br>cephalosporin;penam<br>aminoglycoside<br>acridine_dye;macrolide

					carbapenem;cephalosporin;fluoroquinolone;lincosamide;macrolide;penem;phenicol;rifamycin;tetracycl
**F**	ISEc29<br>ISVsa3<br>ISAba24<br>ISAba26	blaOXA-66<br>blaADC-25<br>blaOXA-23<br>aph(3'')-Ib<br>aph(6)-Id<br>tet(B)<br>armA<br>msr(E)<br>mph(E)	unknown beta-lactam<br>unknown beta-lactam<br>Imipenem;Meropenem<br>Streptomycin<br>streptomycin<br>minocycline, tetracycline, doxycycline<br>minocycline, tetracycline, doxycycline<br>amikacin, gentamicin, tobramycin, isepamicin, netilmicin<br>pristinamycin ia, quinupristin, virginiamycin s, erythromycin, azithromycin<br>erythromycin	abeM<br>adeK<br>adeJ<br>adeI<br>ADC-73<br>A.baumannii_AmvA<br>adeS<br>adeR<br>adeA<br>adeB<br>adeC<br>tet(B)<br>APH(6)-Id<br>APH(3'')-Ib<br>A.baumannii_AbaF<br>OXA-66<br>OXA-23<br>adeN<br>A.baumannii_AbaQ<br>adeH<br>adeF<br>adeG<br>adeL<br>abeS<br>armA<br>msrE<br>mphE	acridine_dye;fluoroquinolone;triclosan<br>carbapenem;cephalosporin;diaminopyrimidine;fluoroq;lincosamide;macrol;penem;phenicol;rifamycin;tet<br>carbapenem;cephalosporin;diaminopyrimidine;fluoroq;lincosamide;macrol;penem;phenicol;rifamycin;tet<br>carbapenem;cephalosporin;diaminopyrimidine;fluoroq;lincosamide;macrol;penem;phenicol;rifamycin;tet<br>cephalosporin<br>acridine_dye;macrolide<br>glycylcycline;tetracycline<br>glycylcycline;tetracycline<br>glycylcycline;tetracycline<br>glycylcycline;tetracycline<br>glycylcycline;tetracycline<br>tetracycline<br>aminoglycoside<br>aminoglycoside<br>fosfomycin<br>cephalosporin;penam<br>cephalosporin;penam<br>carbapenem;cephalosporin;diaminopyrimidine;fluoroq;lincosamide;macrol;penem;phenicol;rifamycin;tet<br>fluoroquinolone<br>fluoroquinolone;tetracycline<br>fluoroquinolone;tetracycline<br>fluoroquinolone;tetracycline<br>fluoroquinolone;tetracycline<br>aminocoumarin;macrolide<br>aminoglycoside<br>lincosamide;macrolide;oxazolidinone;phenicol;pleuromutilin;streptogramin;tetracycline<br>macrolide
**G**	ISEc29<br>ISVsa3	blaOXA-66<br>blaADC-25<br>blaOXA-23<br>aph(3'')-Ib<br>aph(6)-Id<br>tet(B)<br>armA<br>msr(E)<br>mph(E)	unknown beta-lactam<br>unknown beta-lactam<br>Imipenem;Meropenem<br>Streptomycin<br>streptomycin<br>minocycline, tetracycline, doxycycline<br>minocycline, tetracycline, doxycycline<br>amikacin, gentamicin, tobramycin, isepamicin, netilmicin<br>pristinamycin ia, quinupristin, virginiamycin s, erythromycin, azithromycin<br>erythromycin		
**D**	IS6100<br>ISAba13<br>IS26	sul1<br>aph(3')-VIa<br>blaADC-25<br>blaOXA-82<br>aadA2<br>ant(2'')-Ia<br>blaOXA-23	sulfamethoxazole<br>amikacin, neomycin, gentamicin, paromomycin, butirosin, kanamycin, ribostamycin<br>unknown beta-lactam<br>imipenem<br>streptomycin, spectinomycin<br>tobramycin, gentamicin<br>imipenem, meropenem	sul1<br>APH(3')-VIa<br>adeN<br>abeM<br>adeI<br>adeJ<br>adeK<br>A.baumannii_AbaF<br>A.baumannii_AmvA<br>adeC<br>adeB<br>adeA<br>adeR<br>adeS<br>ADC-25<br>adeH<br>adeF<br>adeG<br>adeL<br>abeS<br>OXA-115<br>A.baumannii_AbaQ<br>aadA2<br>ANT(2'')-Ia<br>OXA-23	sulfonamide<br>aminoglycoside<br>carbapenem;cephalosporin;fluoroquinolone;lincosamide;macrolide;penem;phenicol;rifamycin;tetracycl<br>acridine_dye;fluoroquinolone;triclosan<br>carbapenem;cephalosporin;fluoroquinolone;lincosamide;macrolide;penem;phenicol;rifamycin;tetracycl<br>carbapenem;cephalosporin;fluoroquinolone;lincosamide;macrolide;penem;phenicol;rifamycin;tetracycl<br>carbapenem;cephalosporin;fluoroquinolone;lincosamide;macrolide;penem;phenicol;rifamycin;tetracycl<br>fosfomycin<br>acridine_dye;macrolide<br>glycylcycline;tetracycline<br>glycylcycline;tetracycline<br>glycylcycline;tetracycline<br>glycylcycline;tetracycline<br>glycylcycline;tetracycline<br>cephalosporin<br>fluoroquinolone;tetracycline<br>fluoroquinolone;tetracycline<br>fluoroquinolone;tetracycline<br>fluoroquinolone;tetracycline<br>aminocoumarin;macrolide<br>cephalosporin;penam<br>fluoroquinolone<br>aminoglycoside<br>aminoglycoside<br>cephalosporin;penam
**E**	IS6100<br>ISAba13<br>IS26	aph(3')-VIa<br>blaADC-25<br>blaOXA-82<br>aadA2<br>ant(2'')-Ia<br>blaOXA-23<br>sul1	Amikacin<br>unknown beta-lactam<br>Imipenem<br>Streptomycin<br>Gentamicin;Tobramycin<br>Imipenem;Meropenem<br>Sulfamethoxazole	APH(3')-VIa<br>adeN<br>A.baumannii_AbaQ<br>abeM<br>adeI<br>adeJ<br>adeK<br>A.baumannii_AbaF<br>A.baumannii_AmvA<br>adeS<br>adeR<br>adeA<br>adeB<br>adeC<br>adeH<br>adeF<br>adeG<br>adeL	aminoglycoside<br>carbapenem;cephalosporin;fluoroquinolone;lincosamide;macrolide;penem;phenicol;rifamycin;tetracycl<br>fluoroquinolone<br>acridine_dye;fluoroquinolone;triclosan<br>carbapenem;cephalosporin;fluoroquinolone;lincosamide;macrolide;penem;phenicol;rifamycin;tetracycl<br>carbapenem;cephalosporin;fluoroquinolone;lincosamide;macrolide;penem;phenicol;rifamycin;tetracycl<br>carbapenem;cephalosporin;fluoroquinolone;lincosamide;macrolide;penem;phenicol;rifamycin;tetracycl<br>fosfomycin<br>acridine_dye;macrolide<br>glycylcycline;tetracycline<br>glycylcycline;tetracycline<br>glycylcycline;tetracycline<br>glycylcycline;tetracycline<br>glycylcycline;tetracycline

				abeS<br>ADC-25<br>OXA-115<br>aadA2<br>ANT(2'')-Ia<br>OXA-23<br>sul1	fluoroquinolone;tetracycline<br>fluoroquinolone;tetracycline<br>fluoroquinolone;tetracycline<br>fluoroquinolone;tetracycline<br>aminocoumarin;macrolide<br>cephalosporin<br>cephalosporin;penam<br>aminoglycoside<br>aminoglycoside<br>cephalosporin;penam<br>sulfonamide

Table S2. Evaluation of MIC of cloramphenicol (Caf) or ciprofloxacin (Cpx) in the presence of haNPs (8-16 µM albumin concentration) or CCCP (10 µg/ml) as EPI.

Strains	MIC Caf (µg/ml)	MIC Caf + CCCP (µg/ml)	Fold MIC reduction (≥4X)	MIC Caf + haNPs (µg/ml)	Fold MIC reduction (≥4X)
***Ab* A**	256	128	**2**	128	**2**
***Ab* B**	128	32	**4**	256	**0**
***Ab* C**	128	32	**4**	128	**0**
***Ab* D**	128	128	**0**	128	**0**
***Ab* E**	128	64	**2**	128	**0**
***Ab* F**	128	128	**0**	256	**0**
***Ab* G**	128	128	**0**	256	**0**

Strains	MIC Cpx (µg/ml)	MIC Cpx + CCCP (µg/ml)	Fold MIC reduction (≥4X)	MIC Cpx + haNPs (µg/ml)	Fold MIC reduction (≥4X)
***Ab* A**	256	256	**0**	64	**4**
***Ab* B**	64	32	**2**	32	**2**
***Ab* C**	256	32	**4**	128	**2**
***Ab* D**	256	256	**0**	128	**2**
***Ab* E**	256	128	**2**	128	**2**
***Ab* F**	256	128	**2**	128	**2**
***Ab* G**	128	128	**0**	128	**0**

Table S3. MIC (µg/mL), change of MIC and FICI values of Col and haNPs of strains B and G with the checkerboard assay. FICI values: FICI ≤ 0.5, 0.5 < FICI ≤ 1, 1 < FICI ≤ 4, and FICI > 4 were categorized as synergistic (S), additive (A), indifferent (IND) and antagonistic (AN), respectively.

Bacterial strain	MIC$_{col}$ µg/ml	MIC$_{NP}$ (albumin conc.) µM	C$_{col}$ µg/ml	C$_{NP}$ (albumin conc.) µM	Change of MIC$_{col}$	Change of MIC$_{NP}$	FICI	Interaction
A. baumannii strain B	320	> 32	160	0.5	2	> 64	0,515	A
			80	1	4	> 32	0,28	S
			40	2	8	> 16	0,188	S
			20	4	16	> 8	0,188	S
			10	8	32	> 4	0,281	S
			10	16	32	> 2	0,531	A
			5	32	64	> 1	1,016	IND
A. baumannii strain G	80	> 32	1.25	0.5	64	> 64	0,031	S
			0.31	1	258	> 32	0,035	S
			0.156	2	513	> 16	0,064	S
			0.156	4	513	> 8	0,127	S
			0.156	8	513	> 4	0,252	S
			0.156	16	513	> 2	0,502	A
			2.5	32	32	> 1	1,031	IND

Table S4. List of specific primers used to COL-R associated genes

	Sequences of primers (5'-3')	
**Gene**	**Forward**	**Reverse**
adeA	ATCGCTAACAAAGCCTTGAA	CGCCCCCTCAGCTATAGAA
adeB	AACGGACGACCATCTTTGAGTAT	CAGTTGTTCCATTTCACGCATT
adeG	ACTGCAACTTGAAGACCGA	CATCTGTAACAGCAACGCC
adeI	CAAATGCAAATGTAGATCTTGG	AAACTGCCTTTACTTAGTTG
adeJ	GGTCATTAATATCTTTGGC	GGTACGAATACCGCTGTCA
lpxA	AACCACCTACAACCACATGAGAAT	ACCGCCATTATTGATCCATCTGC
lpxC	ACAACACCCGTATCATCTACACCA	ATGAAGTCAGTGAGGCACGAACT
lpxD	TGCTTTCTATGCCTGTTCAGC	CGCTTACATTGTTACCGCAGC
pmrA	GATGGTTTAAATTTGGGTGCAGA	TTGACTCGTAAGTTCAGCTTCT
pmrB	GCCATTATTCGTCGTGGTTTAAA	GCGCTCAAAAAGACGGTTCA
pmrC	TTGCCAAAGATGATGATCGCCCAC	AGCCCTGTATCGCATTCGTATCAC
rpoB	CAAGAGTCTAATGGCGGTGGTTCA	GCGATTGCTTCATCTGCTGGTTG

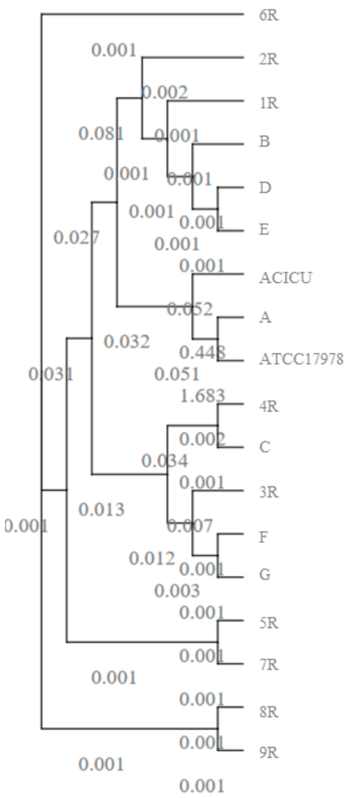

Figure S1. Phylogenetic tree of all clinical *A. baumannii* strains referred to RefGen *A. baumanni* ACICU, realized with the CSI Phylogeny tool.

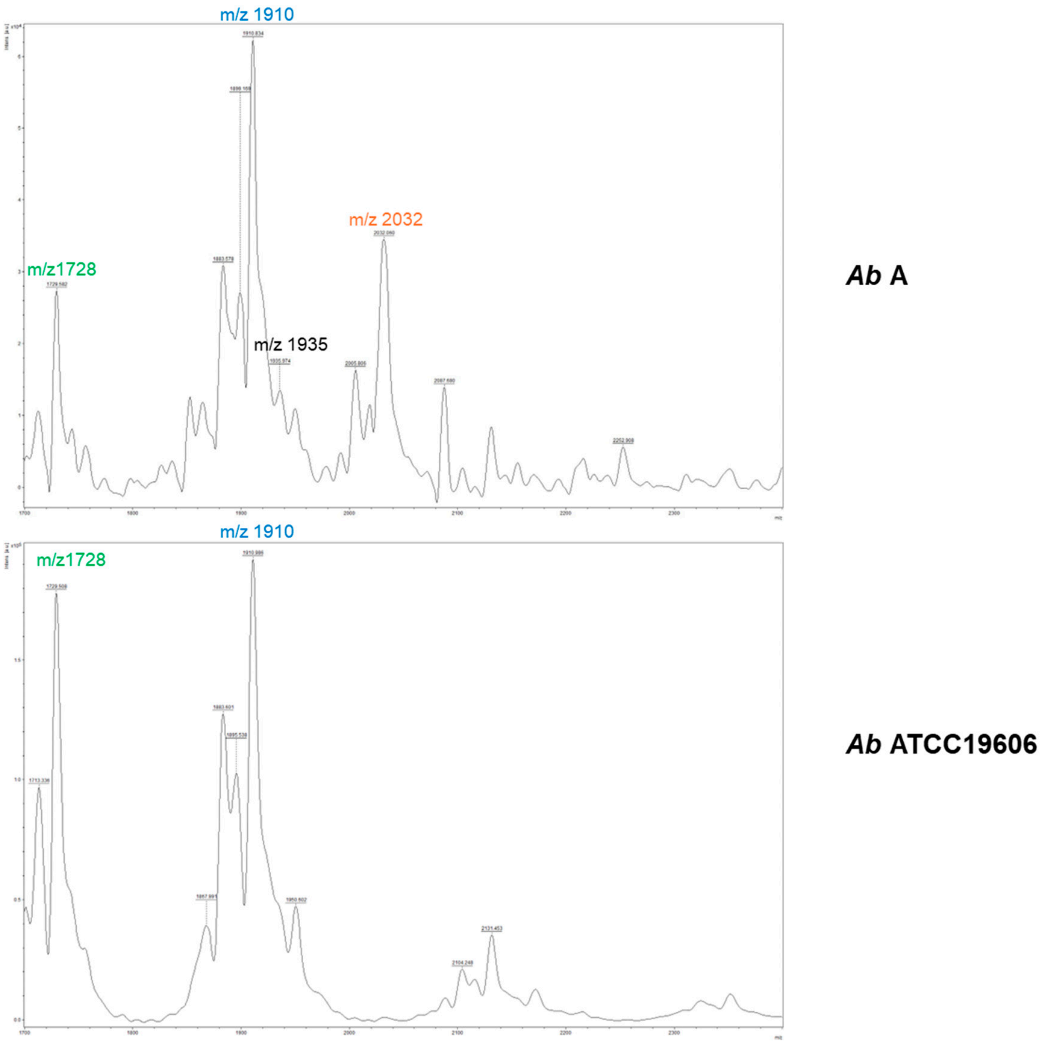

Figure S2. Representative spectra of *A. baumannii* COL-R strain A and *A. baumannii* ATCC19606. Peaks of interest are indicated. The peaks at m/z 1728 m/z and 1910 m/z correspond to native lipid A, the peak at m/z 1935 corresponds to the addition of pETN on the phosphate group at position 4′ of the native lipid A of *A. baumannii* with loss of the phosphate group on position 1, and the peak at m/z 2032 corresponds to the addition of one pETN on the phosphate group of native lipid A.

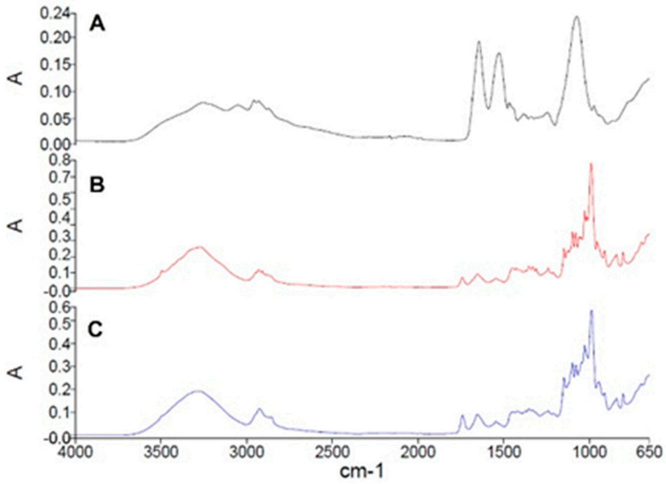

Figure S3. FTIR spectra of Col (A), haNPs (B) and haNPs + Col (C)
